# Stability of di-butyl-dichalcogenide-capped gold nanoparticles: experimental data and theoretical insights†

**DOI:** 10.1039/c9ra07147d

**Published:** 2020-02-10

**Authors:** Luiz Fernando Gorup, Bruno Perlatti, Aleksey Kuznetsov, Pedro Augusto de Paula Nascente, Edison Perevalo Wendler, Alcindo A. Dos Santos, Willyam Róger Padilha Barros, Thiago Sequinel, Isabela de Macedo Tomitao, Andressa Mayumi Kubo, Elson Longo, Emerson Rodrigues Camargo

**Affiliations:** LIEC - Department of Chemistry, UFSCar-Federal University of São Carlos Rod. Washington Luis km 235, CP 676 São Carlos SP 13565-9905 Brazil; Departamento de Química, Campus Santiago Vitacura, Universidad Técnica Federico Santa María Av. Santa María 6400 Vitacura Santiago Chile; Department of Materials Engineering, UFSCar-Federal University of Sao Carlo Rod. Washington Luis km 235, CP 676 São Carlos SP 13565-905 Brazil; Instituto de Química, Universidade de São Paulo São Paulo SP 05508-000 Brazil; Faculty of Exact Sciences and Technology (FACET), Department of Chemistry, Federal University of Grande Dourados Rodovia Dourados, Itahum, Km 12 - Unidade II, Caixa Postal: 364, Cep: 79.804-970 Dourados MS 79804-970 Brazil lfgorup@gmail.com +55 1698100 3030

## Abstract

Metals capped with organochalcogenides have attracted considerable interest due to their practical applications, which include catalysis, sensing, and biosensing, due to their optical, magnetic, electrochemical, adhesive, lubrication, and antibacterial properties. There are numerous reports of metals capped with organothiol molecules; however, there are few studies on metals capped with organoselenium or organotellurium. Thus, there is a gap to be filled regarding the properties of organochalcogenide systems which can be improved by replacing sulfur with selenium or tellurium. In the last decade, there has been significant development in the synthesis of selenium and tellurium compounds; however, it is difficult to find commercial applications of these compounds because there are few studies showing the feasibility of their synthesis and their advantages compared to organothiol compounds. Stability against oxidation by molecular oxygen under ambient conditions is one of the properties which can be improved by choosing the correct organochalcogenide; this can confer important advantages for many more suitable applications. This paper reports the successful synthesis and characterization of gold nanoparticles functionalized with organochalcogenide molecules (dibutyl-disulfide, dibutyl-diselenide and dibutyl-ditelluride) and evaluates the oxidation stability of the organochalcogenides. Spherical gold nanoparticles with diameters of 24 nm were capped with organochalcogenides and were investigated using X-ray photoelectron spectroscopy (XPS) to show the improved stability of organoselenium compared with organothiol and organotellurium. The results suggest that the organoselenium is a promising candidate to replace organothiol because of its enhanced stability towards oxidation by molecular oxygen under ambient conditions and its slow oxidation rate. The observed difference in the oxidation processes, as discussed, is also in agreement with theoretical calculations.

## Abstract 2

This study presents the improved stability against oxidation by molecular oxygen under ambient conditions of organoselenium compared with organothiol, and organotellurium.

## Graphical abstract

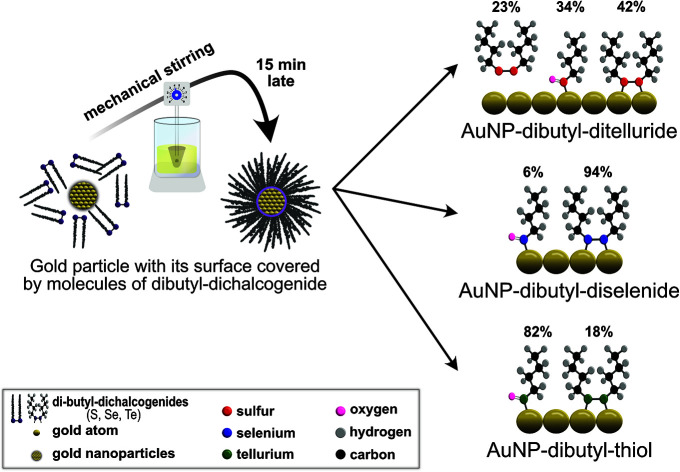

## 1. Introduction

Metal surfaces capped with organochalcogenides form hybrid materials composed of a combination of inorganic components (nanoparticles or films) and organochalcogenides. Their properties are of great interest in various fields, such as surface-enhanced Raman spectroscopy,^1^ catalysis,^2^ sensors,^3^ biosensing and antibacterial agents,^4^ due to their optical and magnetic properties^5^ and their electrochemical,^6^ adhesion,^7^ corrosion and lubrication characteristics.^8^

A popular procedure to obtain these hybrid materials is functionalization of the metal surface with organothiol molecules.^9^ Gold nanoparticles capped by organosulfur compounds on the surface have been well known for decades, and their structures, properties, and applications in diverse fields are still being investigated today. In marked contrast, the chemistry of metal nanoparticles capped with the heavier organochalcogens selenium and tellurium is still an exotic and underdeveloped field of nanotechnology.^10^ Development of synthetic selenium and tellurium compounds has been significant in the last decade; however, it is difficult to find commercial applications for these compounds due to the small number of studies showing the synthetic feasibility and, above all, the advantages of organoselenium and organotellurium compounds over organothiol compounds.

Many properties of these hybrid materials result from strong covalent interactions between the inorganic and organic phases, which is attributed to the strong affinity between the chalcogens (S, Se or Te) and gold, silver or platinum surfaces.^11^ There are numerous reports describing the properties of hybrid materials formed with organothiol derivatives;^12^ however, fewer studies of organoselenium^13^ and organotellurium^14^ have been reported. Recently, one of the authors of this work (A. A. Dos Santos and coworkers) demonstrated the unique characteristics and applications of nanostructured materials based on the synthesis of stable organo-telluro-polymer-capped gold nanostructures; they were found to be stable, dispersible in organic solvents and isolable.^15^ Despite this, there is still a gap to be filled in connection with the properties of previously studied organochalcogenide systems where sulfur is replaced with selenium or tellurium.

As shown by Mekhalif *et al.*,^16^ copper surfaces covered by *n*-dodecaneselenol molecules are less susceptible to oxidation than *n*-dodecanethiol-covered surfaces, showing good blocking properties as well as corrosion inhibition efficiency. On the other hand, Nakamura *et al.*^17^ showed that organotellurium as an anchoring element on the Au(111) surface is more rapidly oxidized when exposed to air compared to an organosulfur-coated surface. In the present study, we perform the first characterizations of organochalcogenide molecules attached to the surface of gold nanoparticles in order to understand the peculiarities of organochalcogenides, more precisely the oxidation stability of the chalcogenide atoms.

In addition, we made the first attempt to compare the properties of three chalcogens, in the molecules dibutyl disulfide, dibutyl diselenide and dibutyl ditelluride, regarding their stability towards the oxidation process. We showed that the organoselenide is generally well-suited to form a compound with high stability to oxidation when compared to tellurium and sulfur. The fraction of oxidized molecules was evaluated quantitatively by X-ray photoelectron spectroscopy (XPS). Based on the results of this analysis, the oxidation stability of the capping molecule monolayers and Au NPs was analyzed. Theoretical calculations were performed as well, and their results corroborated the experimental data. We showed that organoselenium is more stable to the oxidation process than other organochalcogenides; thus, it is a promising candidate to replace organothiols in many applications due to the enhanced stability to oxidation by molecular oxygen under ambient conditions.

## 2. Results and discussion

Organochalcogenides have been targets of interest as intermediates and reagents in organic synthesis because of their synthetic applications and pharmacological properties. Several studies have shown that organic compounds of Se and Te exhibit pharmacological properties as potent therapeutic agents. Therefore, the combination of these molecules with nanoparticles can produce materials of great technological interest.^18^ Organoselenium and organotellurium compounds are very versatile, with numerous opportunities for research, development and applications such as antimicrobial activity,^19^ cancer treatment,^20^ glaucoma treatment,^21^ and catalysis.^22^ S, Se and Te atoms have high affinity for noble metals such as silver, gold and platinum; also, due to their chemical similarity, both tellurium and selenium may eventually replace sulfur in a large number of molecules without increasing their toxicity or causing great environmental impact.^23^

Moreover, in some cases, this substitution can have a profound influence on the chemical properties of hybrid materials, especially when directly related to the covalent bonds between the heteroatoms and metal surfaces.^24^ There is a significant change in the electronic coupling between the heteroatom ligand and metal atoms, affecting the covalent bond energy between the metal and S, Se or Te; advantageously, the surfaces of metal nanoparticles can be modified with organoselenium^22,25^ and organotellurium^26^ molecules which can be prepared by methods similar to those used in the synthesis of organosulfur. To prove the formation of the material, a combination of spectroscopic and microscopic techniques was employed; however, to understand the peculiarities of organochalcogenides, more precisely the oxidation stability of chalcogenide atom, X-ray photoelectron spectroscopy (XPS) studies were performed.

**Fig. 1:**
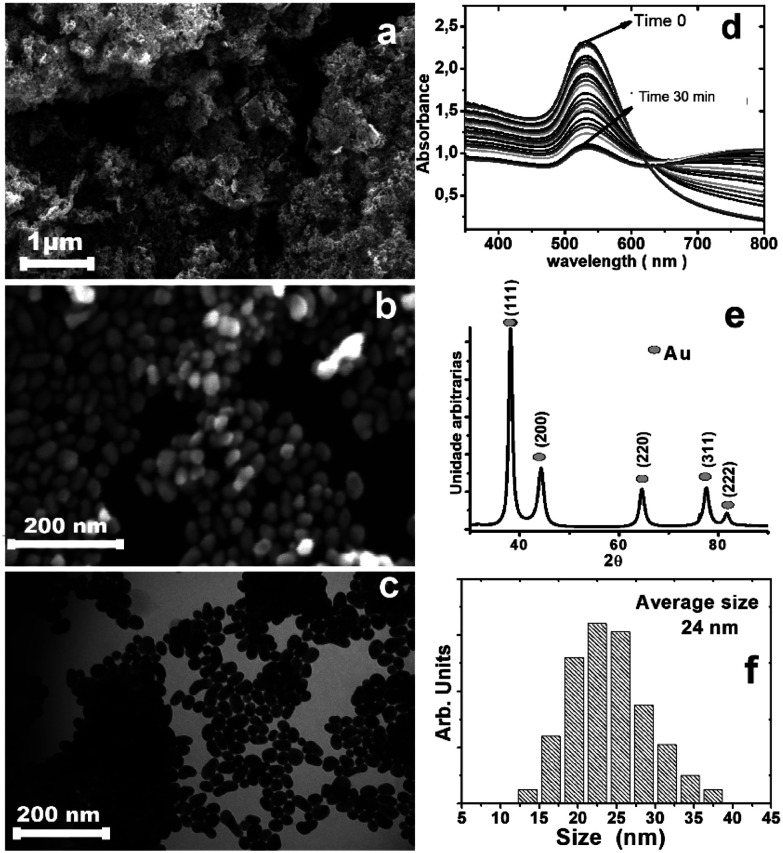
(a and b) Scanning electron microscopy of gold nanoparticles passivated with dibutyl ditelluride on the silicon substrate; (c) transmission electron microscopy of gold nanoparticles passivated with dibutyl ditelluride; (d) UV-vis spectra of the colloidal nanoparticles as a function of time showing decreasing plasmon band intensity, suggesting a continuous decrease of the concentration of the particles in aqueous solution; (e) XRD pattern of the gold nanoparticles covered with dibutyl ditelluride; (f) the particle size distribution of the gold nanoparticles covered with dibutyl ditelluride, where the average size is 24 nm.

The gold nanoparticles were prepared by the citrate method (Fig. S1, ESI†). Dibutyl dichalcogenides were added to the colloidal solution and mixed vigorously; stirring with a vortex favored the formation of bubbles and increased the surface contact between the surface of the nanoparticles and the dibutyl-dichalcogenides. Without stirring the solution, a change in the color of the solution from dark red to dark blue was observed; the intensity decreased with time until the aqueous fraction remained translucent. UV-vis analysis (Fig. 1d) showed the decreased red color intensity of the gold colloid in aqueous solution by absorption of the plasmon peak surface (530 nm), where the colorless state was reached at 30 min. The decrease in the plasmon band at 530 nm is ascribed to a decrease in the number of spheroidal nanoparticles in aqueous solution during formation of the hybrid material because the nanoparticles are not soluble in aqueous solution after coating with organic molecules. The increase of the 800 nm band proves the formation of hybrid material agglomerates, which then can be re-dispersed in non-polar solvents.

The analysis by scanning electron microscopy (Fig. 1a and b) and transition electron microscopy (Fig. 1c) showed 24 nm gold nanoparticles covered by dibutyl ditelluride (Fig. 1f) (see the ESI† for the other dibutyl dichalcogenides). The average particle size did not change after functionalization. The particle with its surface covered by molecules of dibutyl-dichalcogenides loses contact with the solution, and the stability of the nanoparticles in suspension is determined by the physical and chemical characteristics of the molecules bonded on their surfaces. The functionalizing molecule has low solubility in aqueous medium; therefore, after the gold nanoparticles were functionalized, the system became more soluble in polar solvents because the interactions with the solvent molecules were exerted by the functionalizing molecules and not by the surfaces of the nanoparticles.^27^ The samples were deposited on a silicon substrate, dried and stored under ambient conditions of humidity, heat and oxygen. The X-ray, FT-IR, Raman, and XPS analyses were performed with these samples; this was important to evaluate the oxidation stability of the chalcogenide atoms.

Fig. 1e shows the XRD pattern for the obtained material, with peaks at 38.2°, 44.4°, 64.7°, 77.6°, and 81.7° that can be indexed to the (111), (200), (220), (311), and (222) reflections of the face-centered cubic (fcc) structure of metallic Au, respectively (PDF card 04-0784); the particle sizes from the Scherrer formula indicate monocrystalline particles. The calculated cell parameter of 4.115 Å was very close to the value of 4.08 Å found on the PDF card.^28^

The interactions (*i.e.*, charge redistribution) between the anchoring elements and Au core were investigated by analyzing the Au 4f XPS spectra; these were fitted with two doublets, with Au 4f_7/2_ components at 84.2 and 85.8 eV (see Fig. S9†). The lower energy component corresponds to metallic gold atoms located just below the first layer of atoms on the particle surface, while the higher energy component corresponds to gold atoms on the particle surface which are covalently bound to the carboxyl groups of citrate molecules (–CO_2_–) or heteroatom molecules of the organochalcogenides AuX (X = S, Se and Te).

**Fig. 2:**
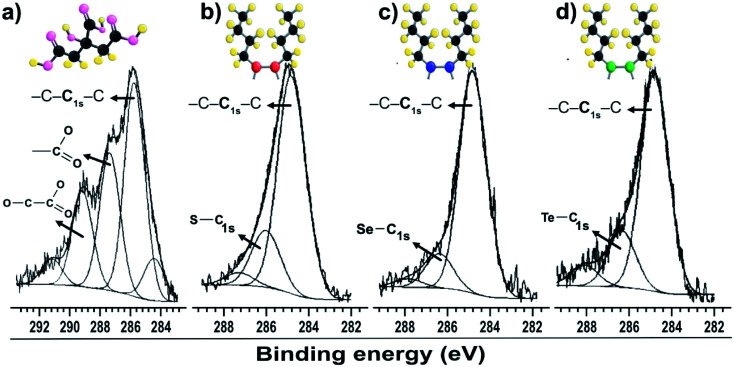
XPS spectra of gold particles with surfaces passivated by (a) citrate C 1s; (b) dibutyl-thiol C 1s; (c) dibutyl-diselenide C 1s; (d) dibutyl-ditelluride C 1s.

The C 1s peaks in Fig. 2b–d were fitted with two components: one at 284.8 eV, corresponding to the C–C and/or C–H chains of aliphatic molecules of dibutyl-dichalcogenide adherent to surface of the nanoparticles, and a less intense component at 286.1 eV, corresponding to carbon covalently bound to the carboxyl group of citrate molecule (–CO_2_–) or an atom of the chalcogenides CX (X = S, Se and Te). A gold colloid sample stabilized only by citrate molecules was analyzed (Fig. 2a). The C 1s peak was decomposed into three components, all corresponding to the adsorption of citrate. The component at lower energy is associated with hydrocarbon species (C–H/C–C; B. E. = 284.5 to 285.7 eV), the component at intermediate energy is associated with carboxyl groups (C 1s with B. E. = 287.4 eV), and the component at higher energy is associated with carboxyl groups (C 1s with B. E. = 289.2 eV), which have the most electropositive carbons due to the proximity of the hydroxyl group.^29^ The XPS measurement results showed that the di-butyl-dichalcogenide-capped gold nanoparticles did not show a significant presence of citrate due to the absence of components reacting with the carboxyl groups. This indicates that the citrate molecules were replaced by di-butyl dichalcogenides on the surface of the gold nanoparticles.

**Fig. 3:**
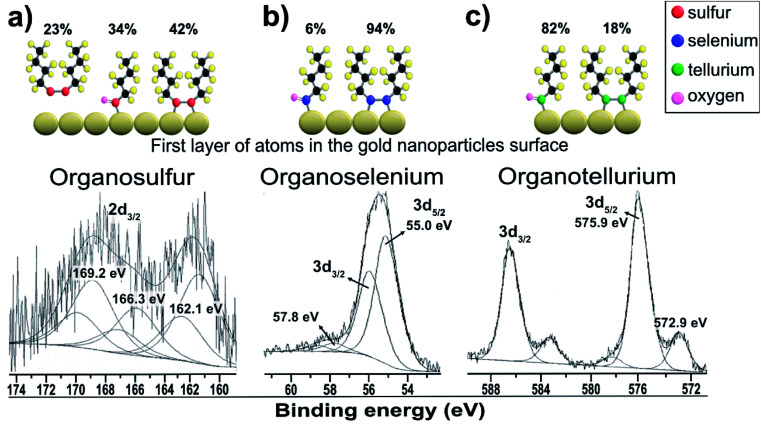
XPS spectra of gold particles passivated with dibutyl-dichalcogenides: (a) dibutyl-thiol S 2p; (b) dibutyl-diselenide Se 3d; (c) dibutyl-ditelluride Te 3d.

**Fig. 4:**
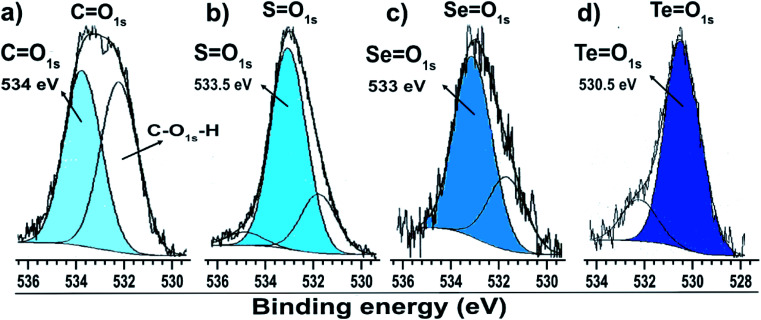
O 1s XPS spectra of gold particles with surfaces passivated by (a) citrate, (b) dibutyl-thiol, (c) dibutyl-diselenol, and (d) dibutyl-ditelluride.

Fig. 3a shows the dibutyl-disulfide S 2p spectrum, which was fitted by three doublets with 2p_3/2_ components at 162.1, 166.3 and 169.2 eV. These indicate the existence of three organothiol species. The S 2p_3/2_ component at approximately 162.1 eV has a higher intensity and is associated with zero oxidation state sulfur, in other words, the dibutyl-disulfide (But_2_S_2_) species. The XPS data indicate that 43% of the total sulfur atoms bound to the gold nanoparticle surface are dibutyl-disulfide species (But_2_S_2_) (Fig. 4b).^30^ The S 2p_3/2_ component with an intermediate energy of 166.3 eV can be associated with a thiol unit; in other words, the component with intermediate energy is associated with a thiol molecule not bound to the nanoparticle surface.^31^ The S 2p_3/2_ component with a higher energy of 169.2 eV can be associated with oxidized species of sulfur (sulfone S=O);^32^ this is corroborated by the presence of the O 1s peak at 533.5 eV (Fig. 4b), indicating the formation of S=O species. The peak areas correspond to the amount of chemical species, and the XPS data indicate that about 34% of the molecules of dibutyl-disulfide underwent oxidation and that 23% of the molecules are not bound to the surface; in this case, one can presume that the sample was not adequately purified and an excess of dibutyl-disulfide molecules was present. The percentage of molecules that underwent oxidation on the surface of the particles is 45%. This oxidation is known and reported in the literature and contributes to greater reactivity of sulfur for oxidation.^33^

Fig. 3b shows the dibutyl-diselenide Se 3d spectrum fitted with two doublets, suggesting the existence of two species of selenium. The two doublets with more intense peaks with components at 55.0 eV (3d_5/2_) and 55.9 eV (3d_3/2_) are associated with selenium, referring to the same chemical state with zero oxidation state. The other, much less intense doublet has components at approximately 57.8 eV (3d_5/2_) and 58.8 eV (3d_3/2_), which are associated with the selenium +2 oxidation state. To identify the proportion of selenium in the two oxidation states, we used the 3d_5/2_ peaks. In the present case, there are two such peaks: one very intense peak at 55.0 eV and another less intense peak at approximately 57.8 eV.

The main 3d_5/2_ peak with a binding energy of 55.0 eV has a peak area of 94% and is associated with the same proportion of selenium with zero oxidation state, that is to say, the dibutyl-diselenide species (But_2_Se_2_) (Fig. 3b). The 3d_5/2_ peak with a binding energy of 57.8 eV has a peak area of 6% and is associated with the same proportion of selenium with the +2 oxidation state, or the oxidized species dibutyl-diselenide (But_2_Se_2_=O).

The low intensity doublet with a 3d_5/2_ component at approximately 57.8 eV can be associated with oxidized species of selenium; this was confirmed by the presence of the O 1s peak at 533.1 eV (Fig. 4c), which indicates the formation of Se=O species. However, it can also be attributed to the Au 5p_3/2_ peak, which has a binding energy approximately equal to 59 eV. The XPS data for the nanoparticles functionalized with organoselenol show that they have greater stability to oxidation compared to the organotellurol and organothiol nanoparticles because only 6% of the selenium atoms underwent oxidation.

Fig. 3c shows the Te 3d dibutyl-ditelluride spectrum; it was fitted with two doublets with 3d_5/2_ components at 572.9 and 575.9 eV, which indicate the existence of two species of tellurium. The less intense component at 572.9 eV is associated with tellurium in the zero-oxidation state, namely the dibutyl-ditelluride (But_2_Te_2_) species. The most intense component at 575.9 eV is associated with oxidized species of tellurium. The difference of 3.0 eV between the peaks indicates the presence of Te(iv), which is confirmed by the presence of the O 1s peak at 530.3 eV (Fig. 4d); this indicates the formation of Te=O species. The obtained results of the latter observation are in agreement with the literature, *e.g.*, Nakamura *et al.*^34^ showed that the XPS spectrum of dibutyl telluride deposited on Au(111) film exhibits peaks of Te 3d_5/2_ at 572.1 and 573.7 eV.

There are reports that indicate that the oxidized species is dibutyl-ditelluroxide (ButTe=O).^35^ This species is also connected to the surface of gold nanoparticles by binding through tellurium. The area of this peak represents the percentage of the chemical species, and the XPS data indicate that about 82% of the molecules of dibutyl-ditelluride underwent oxidation. This oxidation is known and reported in the literature, and it contributes to the greater reactivity of tellurium in oxidation among dibutyl-dichalcogenide molecules.^36^ It is not a good candidate for applications where organotellurol molecules must retain their original properties; however, Nakamura *et al.*^37^ showed that the oxidized species of dioctyl ditelluride remarkably enhance the resistive properties of the OcTe-oxide monolayers, which can lead to effective insulating properties when compared with disulfide and diselenide films.

The XPS analysis suggests that the dibutyl-diselenide molecules bound to the gold nanoparticle surface are more stable towards oxidation processes by oxygen species in air under ambient conditions than the other dibutyl-dichalcogenide molecules (Table 1). Tohru Nakamura *et al.*^17^ suggested that organoselenol and organothiol ligands are more stable than organotellurol ligands, presumably due to the lower oxidation potential of tellurium and the weaker C–Te bond energy than those of the lighter chalcogens (O, S, Se). He showed that tellurophene molecules were gradually oxidized on the surface by oxygen in the atmosphere, faster than their selenium and thiol analogs.

**Table 1:** Stability of dibutyl-dichalcogenide molecules in the oxidation process by oxygen species in air under ambient conditions; percentages of oxidized molecules one day and 13 months after reaction

Organic chalcogenide	Percentages of oxidized molecules ButX=O (X = S, Se or Te)	
	One day after reaction	13 months after reaction
Dibutyl-disulfide	45%	49%
Dibutyl-diselenide	6%	7%
Dibutyl-ditelluride	82%	83%

The three dibutyl-dichacogenide samples were dried after reaction and analyzed by XPS two times, one day and 13 months after sample preparation. The XPS data show that the percentage of oxidized molecules did not change significantly in this period (Table 1); this indicates that oxidation of the chalcogenide occurs during the passivation of the surfaces of the particles, possibly caused by the contact with water. This result suggests a slow oxidation rate for dibutyl-dichalcogenide-capped gold nanoparticles under ambient conditions. The results show that the stability sequence in the oxidation of organochalcogenides is as follows: dibutyl-diselenide, dibutyl-disulfide, and dibutyl-ditelluride. After one year, no significant oxidation of organoselenium was observed; thus, it is a promising candidate to replace organothiol in many applications because of its enhanced stability to oxidation by molecular oxygen under ambient conditions. The observed difference in the oxidation processes of dibutyl-dichalcogenides, as discussed above, is also in agreement with theoretical calculations.^38^

### 2.1. Computational data on the oxidation energies of the capped Au NPs

As models for our theoretical studies, Au_20_ nanoparticles capped either with 1 or 2 X_2_C_8_H_18_ (X = S, Se, Te) ligands, Au_20_(X_2_C_8_H_18_) and Au_20_(X_2_C_8_H_18_)_2_, were used (for discussion of the various DFT and especially molecular dynamics studies of ligand-capped Au NPs with different sizes and for justification of the usage of the Au_20_ model, please refer to the ESI†). To identify the global minima structures of the model compounds, we performed geometry optimizations without any symmetry constraints followed by frequency calculations on both singlet and triplet structures of Au_20_(X_2_C_8_H_18_) and Au_20_(X_2_C_8_H_18_)_2_ using the density functional theory approach.^39^ It was found that for the Au_20_(X_2_C_8_H_18_) species, the singlet structures were lower in energy than the triplets by 8.1 (8.0 with ZPE correction) kcal mol^−1^ for X = S and by 5.4 (4.8 with ZPE correction) kcal mol^−1^ for X = Se. However, for X = Te, the triplet structure was calculated to be lower in energy than the singlet by 6.1 (6.0) kcal mol^−1^ (see the ESI†). Interestingly, for all the Au_20_(X_2_C_8_H_18_)_2_ species, singlets were found to be the lowest-lying structures (ESI†).

In Table 2, the calculated energies of the oxidation reactions of the Au NPs capped with 2 capping ligands, the Au_20_(X_2_C_8_H_18_)_2_ species, are provided. We were interested in studying the oxidation reactions of the Au NPs with higher degrees of surface coverage by the ligands; thus, we decided to focus on the model structures containing 2 ligands. The optimized lowest-lying triplet structures of the Au_20_(X_2_O_2_C_8_H_18_)_2_ species are given in the ESI.†

**Table 2:** Calculated energies of the oxidation reactions of the X_2_C_8_H_18_ (X = S, Se, Te)-capped Au NPs, calculated at the PBEPBE/Lanl2dz level of theory

Δ*E*(Au_20_(X_2_C_8_H_18_)_2_ + O_2_ ⇒ Au_20_(X_2_C_8_H_18_)_2_O_2_), kcal mol^−1^/eV		Δ*E*(Au_20_(X_2_C_8_H_18_)_2_ + 2O_2_ ⇒ Au_20_(X_2_O_2_C_8_H_18_)_2_), kcal mol^−1^/eV	
S	−17.1/−0.74	S	−1.43/−0.062
Se	−21.1/−0.91	Se	−33.48/−1.45
Te	−26.2/−1.14	Te	−51.31/−2.23

For the oxidation of just one of the two capping ligands, the exothermicity of the reaction increased monotonically from S to Te, by 4 kcal mol^−1^ from S to Se and by 5.1 kcal mol^−1^ from Se to Te. That is, the exothermicity of the reaction increases with increasing atomic number of X, as would be expected. However, the situation of the energy differences becomes noticeably different when both capping ligands are oxidized: the exothermicity of the reaction is calculated to change by 32.05 kcal mol^−1^ from S to Se and by 17.83 kcal mol^−1^ from Se to Te. That is, the reaction exothermicity first increases with increasing atomic number of X and then decreases. If we compare the average ligand binding energies calculated for 1 and 2 capping ligands (Fig. S15†), we can see that with 1 capping ligand, the binding energies increase essentially monotonically from S to Te; however, with 2 capping ligands, there is a peak of the ligand binding energies at Se followed by a noticeable drop to Te. A closer look at the optimized structures of the 2 oxidized capping ligands (Fig. S16–S18†) tells us that significant changes in the ligand structures would take place upon oxidation:^40^ breakage of X–X bonds, formation of X–O–X and X–O–Au bridges, and partial removal of ligands from the NP surface with apparent formation of weak van der Waals interactions between the broken ligands and the NP surface or other parts of the ligands (note: a similar situation was observed with the relatively long-chain SCH_2_CO_2_H ligands in the 2014 study of capped Cd_33_X_33_ (X = Se, Te) NPs performed by Kuznetsov and Beratan^41^). Therefore, the process of the capped ligand oxidation is very complicated, and many different factors, both thermodynamical (relative stabilities and reaction energies) and kinetic, should be taken into account when considering it. We can see that introduction of a second ligand in the models changes the ligand binding energies (see Fig. S15†); thus, the capped NPs with 2 S-containing ligands are more stable towards dissociation to the bare NPs and free ligands than the capped NPs with 2 Te-containing ligands, and the NPs with 2 Se-containing ligands are the most stable of all the three species. However, these relative stabilities should not be confused with the stabilities towards oxidation. According to previous studies (see the ESI†), coverage of Au NPs with Se-containing ligands increases their stability against degradation in solution^42^ (ref. 32 in the ESI†); however, upon coverage of the Au NPs with the Te-containing ligands, it is difficult to completely prevent the oxidation of tellurolate in the nanoparticles^43^ (ref. 33 and 34 in the ESI†). We suppose that increasing the ligand binding energies from the S-containing to Se-containing ligands opposes the increase of the exothermicity of the oxidation reaction from S to Se, and, in contrast, the significant decrease of the ligand binding energies from Se to Te opposes the decrease in the exothermicity of the oxidation reaction from Se to Te. Also, we suppose that mutual interactions of the ligands closely located at the NP surface and various changes in the ligand structures upon oxidation may change the reactivity of the ligand towards O_2_. We believe that complete understanding of the oxidation of Au NPs capped with ligands containing three different chalcogens will require further detailed theoretical and experimental investigations. However, we believe that we have provided explanations for the current experimental findings.

## 3. Experimental section

### 3.1. Synthesis of gold nanoparticles

**Fig. 5:**
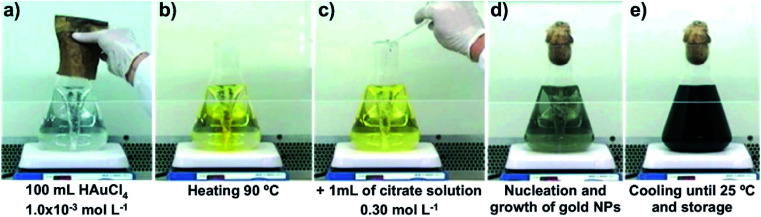
Synthesis of gold nanoparticles by the standard citrate method.

Gold nanoparticles were prepared by the reduction of gold(iii) chloride trihydrate (99.9%, Sigma-Aldrich, USA) solutions with sodium citrate (99% Synth, Brazil) by the standard procedure, following the citrate method.^44^ Solutions were prepared with deionized water obtained from a commercial Millipore Elix 3 system. All the chemicals used in this work were of analytical grade and were used as received with no further purification. A volume of 100 mL of an aqueous solution of HAuCl_4_ (1.0 mmol L^−1^) was heated and stirred gently with a magnetic Teflon-coated bar. When the temperature of the HAuCl_4_ solution reached 90 °C, 1.0 mL of a 0.3 mol L^−1^ solution of sodium citrate preheated to 90 °C was added, and the pH of the mixture, measured at room temperature, was maintained at 3 to 4. After 8 minutes of reaction, the reaction mixture was removed from the heat and cooled to room temperature (Fig. 5).

### 3.2. Synthesis of dibutyl-dichalcogenides

#### Chemicals

Tellurium (200 mesh, 99.85%), selenium, sulfur, sodium borohydride (powder, 98%), bromine, iodine, *n*-butyl lithium solution, and 1-bromobutane (BuBr, 99%) were purchased from Aldrich. Sodium sulfite was purchased from EMScience. Tetrabutylammonium bromide (TBAB, 98%) was obtained from Alfa Aesar, and hydrogen tetrachloroaurate hydrate (49 wt% Au) was obtained from Strem Chemicals. The organic solvents dimethyl formamide, hexane, toluene, methanol, dichloromethane, ethanol, and tetrahydrofuran were of analytical grade. They were all used as received. Milli-Q water (18.2 MΩ) was used. All glassware was cleaned in sulfuric acid with Nochromix and rinsed with a large amount of water before use.

**Fig. 6:**
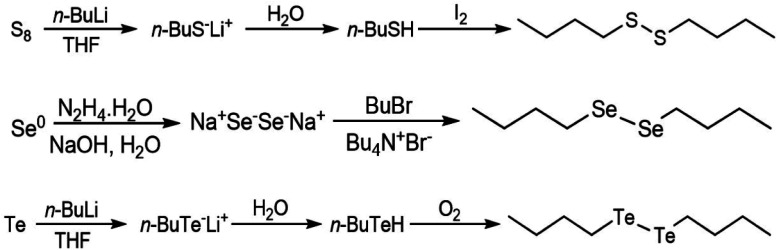
Schemes of the syntheses of the dibutyl-dichalcogenides.^45^

Di-butyl disulfide was prepared by addition of *n*-butyl lithium (1 eq.) to a THF suspension of elemental sulfur followed by water and iodine. A similar procedure was adopted to prepare dibutyl ditelluride, omitting the treatment of the reaction media with iodine and exposing the intermediate tellurol to an oxygen atmosphere for the oxidation step. Di-butyl diselenide was prepared by reacting elemental selenium in aqueous basic media with hydrazine hydrate followed by addition of butyl bromide and tetrabutylammonium bromide. The synthetic reaction is summarized in Fig. 6. A detailed experimental procedure is presented in the ESI.†

### 3.3. Synthesis of hybrid materials

The hybrid materials were prepared by anchoring dibutyl-dichalcogenide molecules on the surface of gold nanoparticles (Fig. 3). The colloidal gold nanoparticles used were obtained after eight minutes of reaction. The organic molecules of dibutyl-dichalcogenides were provided, synthesized and purified by the Laboratory of Organocatalytic and Synthesis of Bio-active Substances (ESI†).

Three samples of hybrid materials were prepared simultaneously in duplicate; the three species of dibutyl-dichalcogenides used to passivate the surfaces of the gold nanoparticles were dibutyl-disulfide, dibutyl-diselenide, and dibutyl-ditelluride. 45 mL of gold colloid were mixed in a falcon tube with 5 mL of the functionalizing agent (organochalcogenide) solution (with a concentration of 45 mmol L^−1^ in chloroform). The mixture was stirred vigorously with a vortex (IKA® Vortex VG 3.35) at the number 4 position of the rotating knob. After 15 minutes of vigorous stirring, the color of the colloid changed from dark red to a dark blue tint, which lost intensity with time until the aqueous fraction remained translucent. The vigorous stirring of the solutions with a vortex favored the formation of bubbles and increased the surface contact between the surface of the particles and the dibutyl-dichalcogenides.

**Fig. 7:**
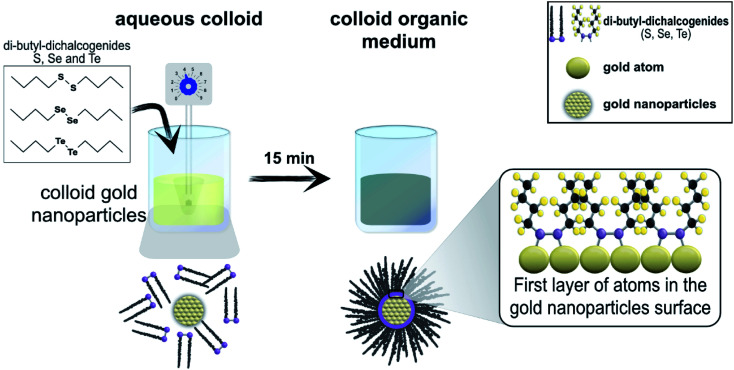
Scheme of the synthesis of the hybrid materials (nanoparticles + dibutyl-dichalcogenide molecules).

The functionalized nanoparticles formed a film in the organic phase. The organic phase with functionalized particles was collected, isolated by centrifugation (3000 rpm for 10 minutes) and washed several times with chloroform to remove excess functionalizing agent (Fig. 7). All syntheses were repeated at least twice, and the results were always reproducible. In order to standardize the functionalization, a 5 : 1 ratio of the functionalizing agent to the initial concentration of ions in the colloid was chosen. This stoichiometric relationship is apparent because it does not represent the stoichiometric relationship between the active sites and the functionalizing agent. Considering that particles with diameters of 24 nm have approximately 7% of their atoms on the surface, which are the active anchoring sites for the organochalcogenides, the stoichiometry of the functionalizing agent and active sites is 71 : 1.

Three samples with the three different dibutyl-dichalcogenide molecules were also prepared where the functionalization was processed without agitation. For the functionalization of the nanoparticles with the organochalcogenides, 5 mL of gold colloid were added to a quartz cuvette; then, 50 μL of functionalizing agent (0.10 mol L^−1^) of the dibutyl dichalcogenide were added. The UV-vis spectra of each resulting solution were collected every minute in the range of 0 to 99 minutes. After one hundred minutes, the gold particles were collected and sedimented, washed (5×) with chloroform and analyzed by scanning electron microscopy.

### 3.4. Characterization

#### DRX

Gold nanoparticles were characterized in the 2*θ* range from 20 to 110° by X-ray diffraction (XRD) using a Rigaku D/max 2500PC diffractometer with Cu Kα radiation operating at 40 kV and 40 mA. To collect the patterns, the nanoparticles were deposited on a silicon substrate by dripping the aqueous colloidal dispersion onto the substrate at room temperature and waiting for the solvent to evaporate.

#### TEM-MEV

Scanning transmission electron microscopy (STEM) images were recorded at 20 kV using a FEG Zeiss Supra 35-VP, and scanning electron microscopy (SEM) images were recorded with a Zeiss DSM 940A. The samples were prepared by placing two or three drops of the dilute NP dispersion in water on carbon-coated copper grids (200 mesh, PELCO® Center-Marked Grids). Histograms were constructed using the public domain ImageJ image processing software.

#### IR

The IR spectra were obtained using a Bruker infrared spectrometer. For the free Oct2Te2, the liquid sample was dropped onto a KBr pellet and covered with another KBr pellet. For the Au NPs, a small amount of NPs in solid form was mixed with KBr powder as a solid support and ground to an even mixture with a pestle. The mixture was pressed into a wafer for IR measurement. All measurements were performed in transmittance mode at 2 cm^−1^ resolution. The spectra were background subtracted with respect to KBr and subjected to baseline correction and smoothing.

#### UV-vis

For the UV-vis spectroscopy, colloidal gold was transferred into a 1.0 cm path length quartz cuvette, and the measurements were performed on a Shimadzu Multspec 1501 UV-vis spectrometer. A typical experiment scanned the wavelength range from 275 to 800 nm. Background adjustments were made using water as a blank.

#### XPS

X-ray photoelectron spectroscopy (XPS) was used to assess the monolayer elemental composition and oxidation states of sulfur, selenium and tellurium atoms residing at the gold nanoparticle surface. XPS analyses were performed using a Kratos XSAM HS spectrometer with energy resolution greater than 0.1 eV. The analyses were carried out in an ultrahigh vacuum atmosphere (10^−8^ Torr) using magnesium Kα radiation as the excitation source, with an energy of 1253.6 eV and 50 W power provided by 10 kV voltage and 5 mA emission. A reference binding energy of 284.8 eV was used for the C 1s peaks associated with C–C and/or C–H.^46^

Peak fittings were performed using Gaussian curves (for the C 1s and O 1s peaks) and Gaussian/Lorentzian curves (for the Se 3d and Te 3d doublets), background removal by the Shirley method, and the least-squares routine.^47^ The samples were deposited on Si (111) with an area of 1.5 cm × 1.5 cm and mounted using conductive copper-backed double-sided adhesive carbon conductive tape (PELCO Tabs™, 16073). The sensitivity factors for quantitative analysis were referred to *S*_F 1s_ = 1.0. In the cases of alkanethiols, alkaneselenols and alkanetellurols, the C 1s line mainly consists of carbon atoms from methylene groups that all have the same chemical environment (with the exception of the C atom bound to the head group); this line appears as a simple and well-defined structure in the spectra.

## 4. Conclusions

We report here the successful synthesis of hybrid materials with gold nanoparticles and a series of dibutyl-dichalcogenides (S, Se and Te). The average particle size of the formed Au NPs measured by transmission electron microscopy (TEM) was 24 nm, and XPS was used to investigate the composition and structure of the capping monolayers and the formed Au NPs. We showed that organoselenium is more stable to the oxidation process than other organochalcogenides. After one year, no significant oxidation of organoselenium was observed; thus, it is a promising candidate to replace organothiol in many applications because of its enhanced stability to oxidation by molecular oxygen under ambient conditions. These characteristics were observed here for the first time. Theoretical calculations were performed, and their results supported the experimental results. Our future plans regarding these systems include studies of the kinetics of the autoxidation process and whether the structural quality of the films is fully preserved after autoxidation. In addition, it would be interesting to examine in more detail the initial stages of autoxidation of the organochalcogenides which are readily oxidized by oxygen species under ambient conditions, presumably by hydrogen oxide in the reaction solution rather than oxygen gas.

## Conflicts of interest

There are no conflicts to declare.

## Supplementary Material

RA-010-C9RA07147D-s001
