# Supplementary material for: Stability of di-butyl-dichalcogenide-capped gold nanoparticles: experimental data and theoretical insights
Source: RSC Adv. 2020 Feb 10;10(11):6259–70. doi: 10.1039/c9ra07147d (PMC9049692; doi:10.1039/c9ra07147d)
Supplement: RA-010-C9RA07147D-s001 [file RA-010-C9RA07147D-s001.pdf]

Electronic Supplementary Material (ESI) for RSC Advances.
This journal is © The Royal Society of Chemistry 2019

SUPPORTING INFORMATION

1.EXPERIMENTAL PROCEDURE

Solutions were prepared with deionized water obtained from a commercial Millipore Elix 3 system. All the chemicals used in this work were of analytical grade and were used as received, with no further purification. gold nanoparticles were prepared by the reduction of $HAuCl_4$ (98.8% Merck, Germany) solutions with sodium citrate (99% Synth, Brazil) by the standard method described by Turkevich. A volume of 100 mL of an aqueous solution of $HAuCl_4$ (1.0 mmol L^{-1}) was heated and stirred gently with a magnetic Teflon-coated bar. When the $HAuCl_4$ solution reached 90°C, 1.0 mL of a 0.3 mol L^{-1} solution of sodium citrate preheated to 90°C was added and the pH of the mixture, measured at room temperature, was kept at 3,4. During the reaction, aliquots of 10 mL were removed from the flask, cooled to room temperature and subjected to UV-Vis characterization to monitor the reduction of gold ions based on the characteristic plasmon absorbance band at approximately 530 nm.

Gold nanoparticles were characterized in the 2θ range from 20 to 110° by X-ray diffraction (XRD) using a Rigaku Dmax 2500PC diffractometer with CuKα radiation operating at 40 kV and 40 mA. To collect the patterns, nanoparticles were deposited on silicon substrate by dripping the aqueous colloidal dispersion onto the substrate at room temperature and waiting for the solvent to evaporate. The UV-Vis spectra of gold nanoparticles aqueous dispersions were obtained with a UV-Vis spectrophotometer (Shimadzu Multspec 1501) in the region of 190 to 800 nm, using a commercial quartz cuvette. Scanning transmission electron microscopy (STEM) images were recorded at 20 kV using a FEG Zeiss Supra 35-VP and scanning electron microscopy (SEM) images were recorded with a Zeiss DSM 940A. Histograms were constructed using the public domain ImageJ image processing software. To estimate the reaction yield, conductometric titrations were performed at room temperature.

2. SYNTHESIS OF GOLD NANOPARTICLES

Figure SI.1 shows a series of gold colloids synthesized at pH 3,4 and 90°C as a function of reaction time, as indicated by the numbers in minutes. It is evident that with longer times the colloids become red, indicating high concentrations of nanoparticles, while in shorter times, the colloids are red because of the plasmon band of gold nanoparticles formed. It is important to note that after 8 minutes the red color does not

change. A red coloration and the position of maximum absorption at 530 nm observed in the UV-Vis spectrum of Figure SI-1 indicates particles of about 24 nm in good agreement with the TEM image, The X-ray diffraction pattern (XRD) confirms that the gold nanoparticles have a cubic crystal structure of face-centered and diffraction peaks are consistent with the average particle size observed in the TEM image of 24 nm.

XRD pattern of spherical Au nanoparticles. Four peaks observed at 38.2°, 44.4°, 64.7°, 77.6°, and 81.7°can be indexed to the (111), (200), (220), (311), and (222) reflections of face-centered cubic (fcc) structure of metallic Au, respectively (PDF card 04-0784), showing the crystalline particles. and the calculated cell parameter of 4.115 Å was very close to the value of 4.08 Å found on the PDF card.

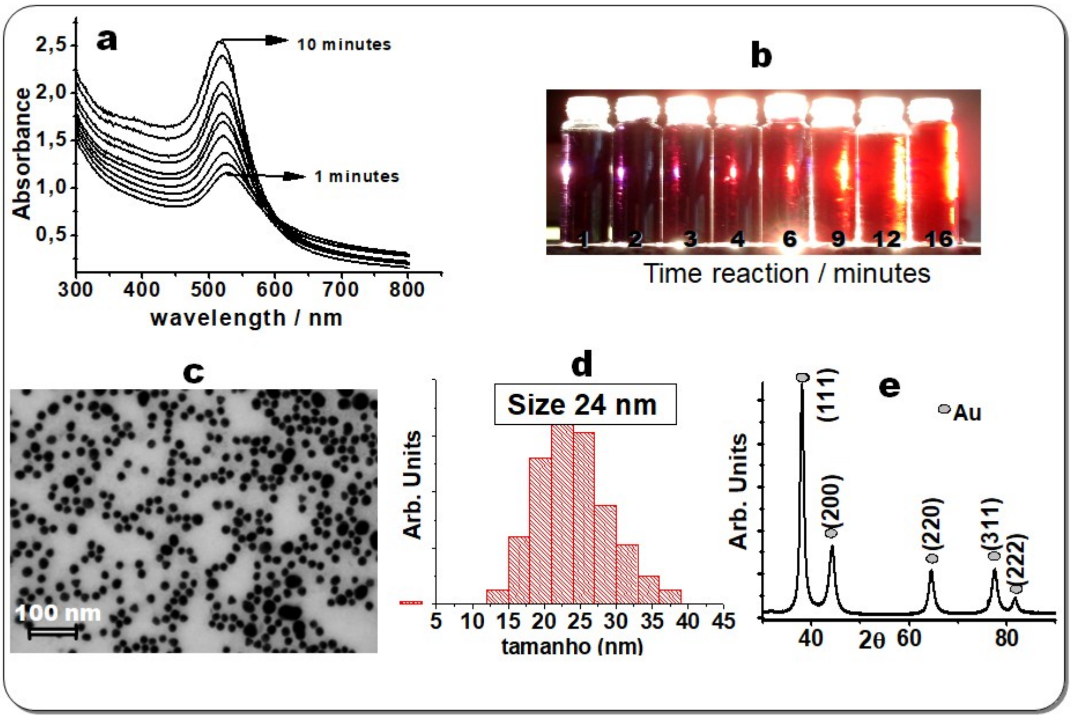

Figure SI.1: Gold colloids synthesized at pH 3,4 and 90ºC as a function of the reaction time in minutes. The red color indicates the presence of gold nanoparticles and the absorbance is proportional to the nanoparticle concentration. a) UV–Vis spectra of the colloidal nanoparticles in time 0 to 16 minutes with expressive increasing of the plasmon band intensity, suggesting the continuous increase of concentration of particles in the aqueous solution; b) Image of colloidal nanoparticles with color purple for one minute of reaction and red color after 6 to 16 minutes; c) Transmittion electron microscopy of gold nanoparticles; d) The average size of the gold nanoparticles with size 24 nm. e) XRD pattern of gold nanoparticles.

3. SYNTHESIS OF DIBUTYL DICHALCOGENIDES

Dibutildisulfide[1]:

$$S_8 + BuBr \xrightarrow[THF]{n\text{-}BuLi} BuS^{-}Li^{+} \xrightarrow{H_2O} BuSH \xrightarrow{I_2} BuSSBu$$

To a 25mL round bottom flask was added 64,2mg (0,25mmol) of elemental sulphur, in its orthorhombic form containing 8 sulphur atoms, in N_2 atmosphere. 2mL of freshly distilled THF was added, and the solution was stirred with a magnetic stirrer. n-Butyllithium (2,62mL, 2 mmol) was added dropwise with continuous stirring, using an ice bath to control the reaction temperature, until the solution got nearly colorless, and 0,06mL (3mmol) of distilled water was added as a source of protons. In other flask, 0,508g (2mmol) of iodine was placed, and 2mL of THF was added, under N_2 atmosphere and manual agitation. The iodine solution was then transferred to the reaction, until the iodine color started to prevail over the solution. The reaction was filtered through a pad of silica on a sintered plate funnel, and extracted with hexane (2x10mL). The organic layer was separated and washed with distilled water (1x10ml) and brine (2x5mL). The organic layer was dried with $MgSO_4$, filtered and the solvent evaporated to give a yellow liquid, in 64% yield.

Dibutilditelluride[2] :

$$Te^0 + BuBr \xrightarrow[THF]{n\text{-}BuLi} BuTe^{-}Li^{+} \xrightarrow{H_2O} BuTeH \xrightarrow{O_2} BuTeTeBu$$

To a 40mmol suspension of Te in THF (50 mL) under nitrogen at 0ºC, 1 equivalent of 2M n-BuLi in hexane was added. The mixture was stirred for 35 min at 0°C and 15 min at r.t.. The mixture was added to a flask containing water (100 mL), and stirred for another 15 min. The organic phase was separated, washed with distilled water (200 mL) and brine (200 mL), dried with MgSO4, filtered and evaporated to give a dark red oil, in 92% yield.

Dibutildiselenide[3]:

$$Se^0 \xrightarrow[NaOH,\ H_2O]{N_2H_4.H_2O} NaSeSeNa \xrightarrow[Bu_4^-N^+]{BuBr} BuSeSeBu$$

To a 100mL round bottom flask containing 4,345g (55mmol) of selenium powder and 2,2g (55mmol) of NaOH, 20mL of distilled water was added, under nitrogen atmosphere and magnetic stirring. 1,57mL (19mmol) of 80% hydrazine hydrate was added during 30 minutes, and the reaction was left stirring at 70ºC for about 3 hours, until all the elemental selenium had dissolved. The solution was taken out of the heat, and when it got back to ambient temperature 5,37mL (50mmol) of 1-bromobutane and 0,516g (1,6mmol) of tetrabutylammonium chloride was added, causing the separation of a light-red oil. The layers were separated, and the aqueous layer was extracted with hexane (2x20mL). The combined organic phases were washed with distilled water (2x15mL) and brine (2x15mL), dried with $MgSO_4$ and the solvent evaporated to give a orange oil, in 79,4%.

4. SYNTHESIS OF HYBRID MATERIALS

The hybrid materials were prepared by anchoring of dibutyl-dichalcogenides molecules on the gold nanoparticles surface (Fig. SI-2). The colloidal gold nanoparticles used were those obtained after eight minutes of reaction.

Organic molecules of dibutyl-dichalcogenides used were provided, synthesized and purified by Laboratory of Organocatalytic and Synthesis of Bio-active Substances.

Three samples of hybrid material were prepared simultaneously in duplicate, the three species of dibutyl-dichalcogenides used to passivate the surfaces of the gold nanoparticles were dibutyl-dithiol, dibutyl-diselenol, dibutyl-ditellurol. 45 mL of gold colloid were mixed in a falcon tube with 0.45 mL of the functionalizing agent (organochalcogenide) with concentration of 45 mmol L-1 with 5 mL of chloroform. The mixture was stirred vigorously with a vortex (IKA ® Vortex VG 3.35) in the 4 number position of rotating knob.

After 15 minutes of vigorous stirring, color (?) of the colloid changed from dark red to a dark blue tint and was losing the intensity with time until the aqueous fraction stayed translucent. The vigorous stirring of solutions with a vortex favored the formation of bubbles and increased the surface contact between the surface of particles and dibutyl-dichalcogenides.

The functionalized nanoparticles formed a kind of film involving the organic phase. The organic phase with functionalized particles was collected and isolated by

centrifugation (3000 rpm for 10 minutes) and washed several times with chloroform to remove excess functionalizing agent. All syntheses were repeated at least twice, and the results were always reproducible.

In order to standardize functionalizing the ratio 5:1 between the functionalizing agent and initial concentration of ions in the colloid, respectively, was chosen. This stoichiometric relationship is apparent, because it does not represent the stoichiometric relationship between active sites and functionalizing agent.

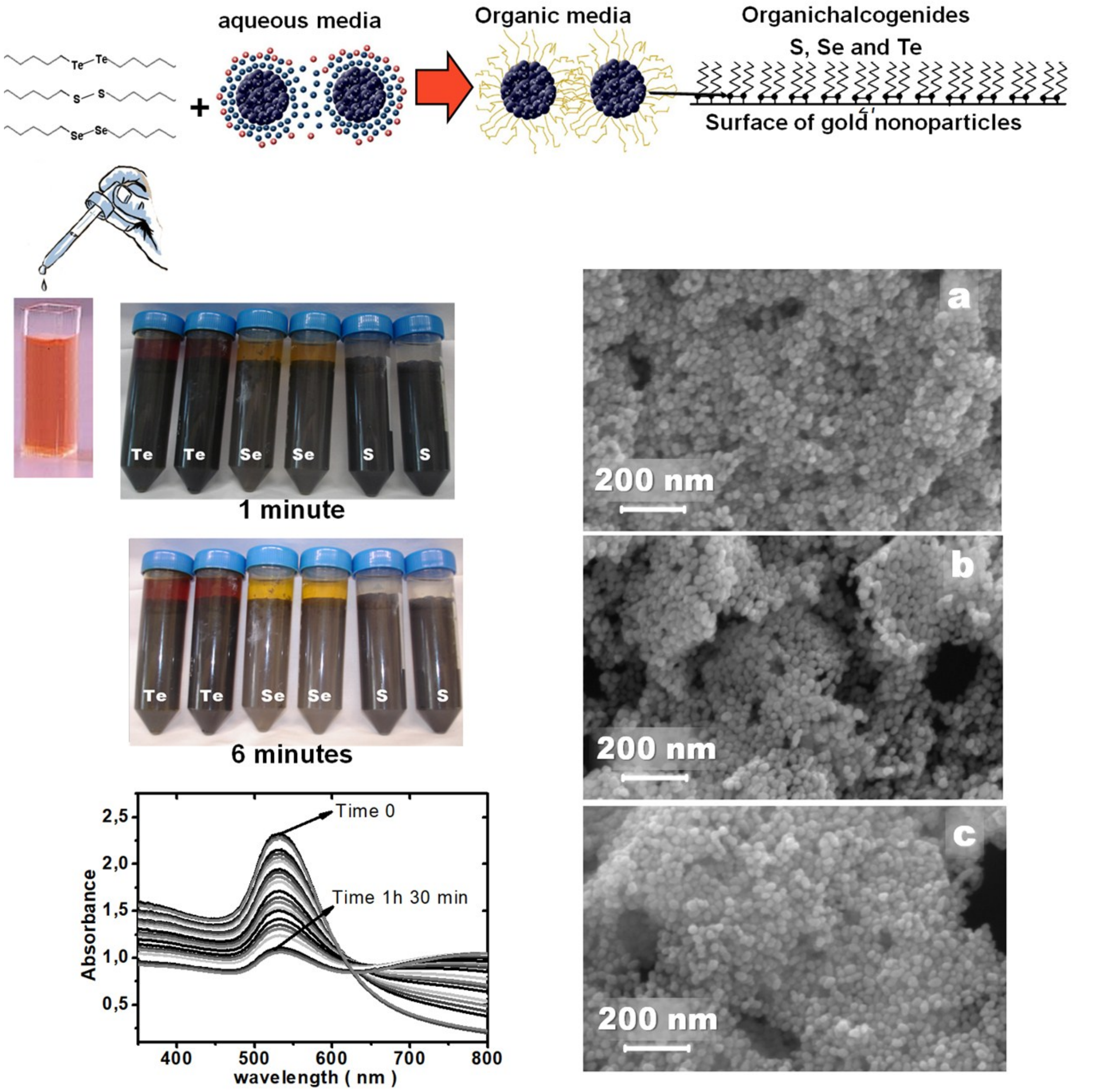

Figure SI.2: (a) UV-Vis spectra of the gold colloids functionalization as a function of time. The red color, which indicates the presence of gold nanoparticles, remained almost constant over time. **a-c)** Scanning and transmission electron microscopy of gold nanoparticles passivated with dibutyl ditellurol on the silicon substrate; d) UV–Vis spectra of the colloidal nanoparticles in time 0 to 30 minutes with expressive decreasing of the plasmon band intensity, suggesting the continuous decrease of concentration of particles in the aqueous solution.

Considering that particles with diameter of 25 nm have approximately 7% atoms on the surface of particles and only the surface atoms are sites for the anchoring of the molecules organichalcogenides, the stoichiometric of functionalizing agent and active sites is 71:1.

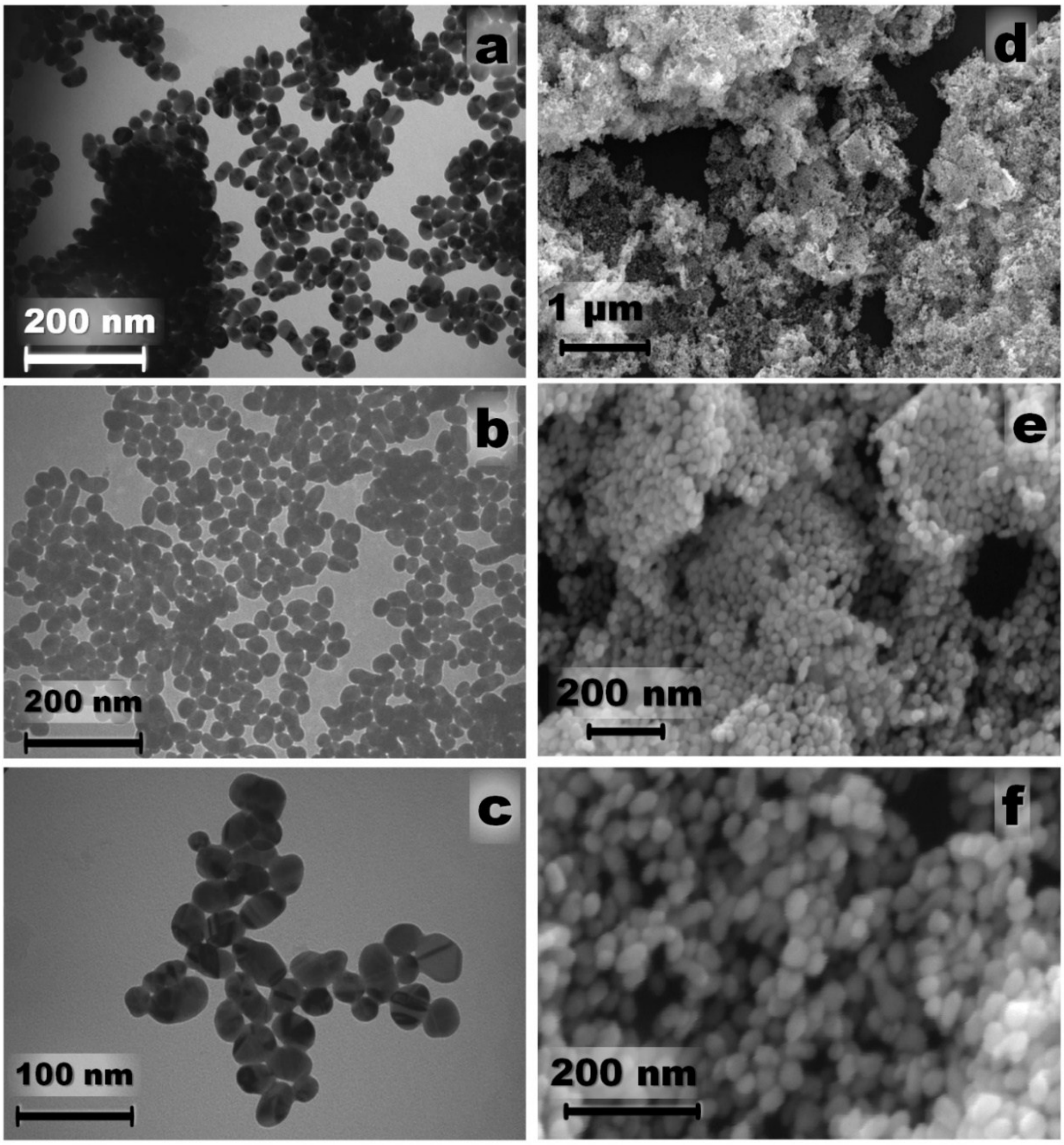

Figure SI.3: Gold nanoparticles passivated with dibutyl ditellurol. **a-c) T**ransmission electron microscopy; **d-f)** Scanning electron microscopy

The analysis by scanning and transition electron microscopy (Fig.SI 2 and 3) showed gold nanoparticles with size 24 nm (Fig.4f). The average particle size was not changed after functionalization. The particle with its surface covered by molecules of dibutyl-dichalcogenides loses contact with the solution and the stability of nanoparticles in suspension is determined by physical and chemical characteristics of molecules bonded on its surface.

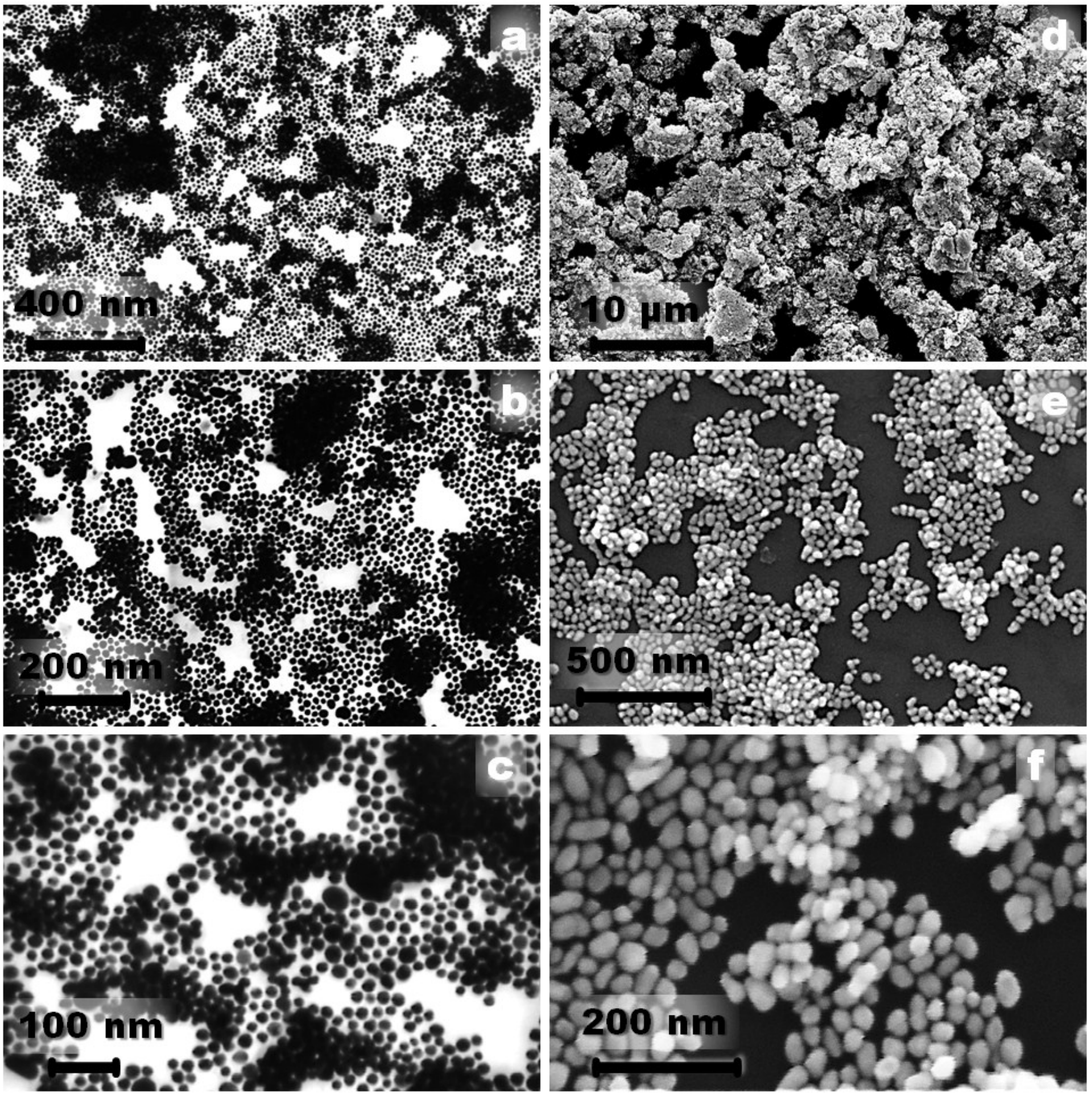

Figure SI.4: Gold nanoparticles passivated with dibutyl selenol. **a-c)** **T**ransmission electron microscopy; **d-f)** Scanning electron microscopy

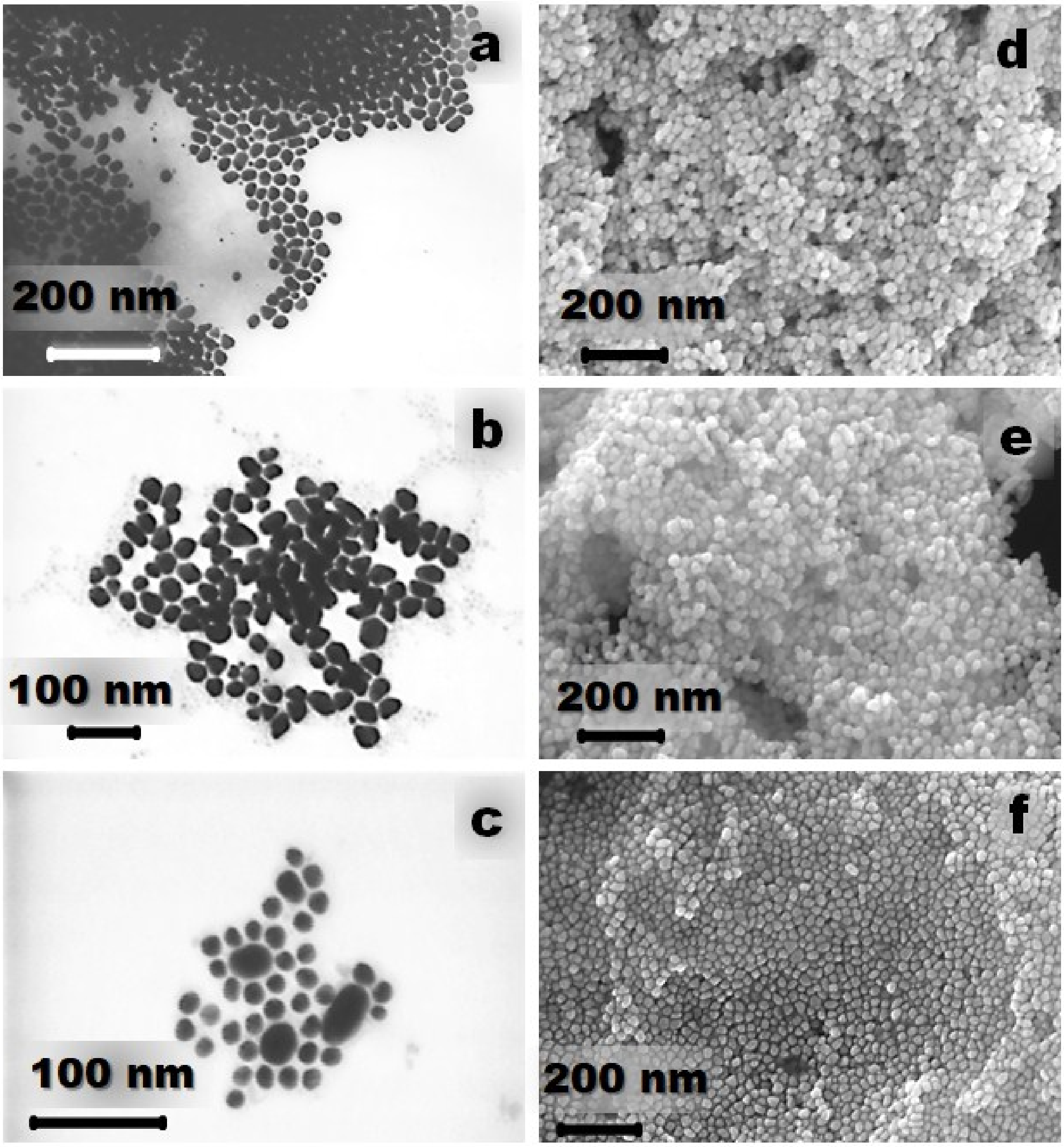

Figure SI.5: Gold nanoparticles passivated with dibutyl dithiol. **a-c)** **T**ransmission electron microscopy; **d-f)** Scanning electron microscopy

4. RAMAN AND IR ANALYSIS DIBUTYL-DICHALCOGENIDES MONOLAYES

The figure S4-6 and b displays IR spectra of dibutyl-dichalcogenides monolayers on surface gold nanoparticler. In the alkyl stretching mode region (2800–3000 cm^{-1}), two bands appear at 2922 and 2851 cm^{-1} corresponding to the mas(CH2) and ms(CH2)modes, respectively; the Fermi resonance of ms(CH2) + 2d(CH2) is located at 2894 cm^{-1}. We note that the position of the mas(CH2) and ms(CH2) vibration modes indicates that the alkyl chains are crystal-like as compared with the corresponding peaks centered at 2920 and 2850 cm^{-1} for crystalline all-trans polymethylene.

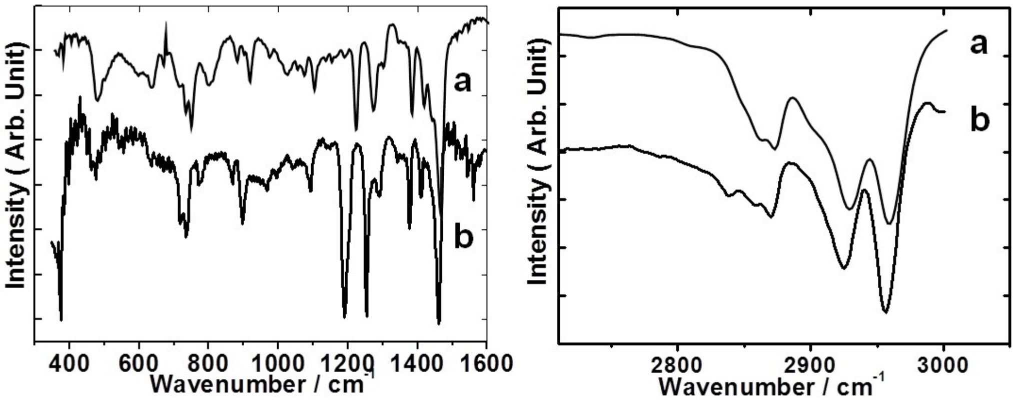

Figure SI-6: IR spectrum of dibutyl-thiol. a) the dibutyl-thiol dropped in silicon substrate; b) the dibutyl-thiol linked in gold particles surface.

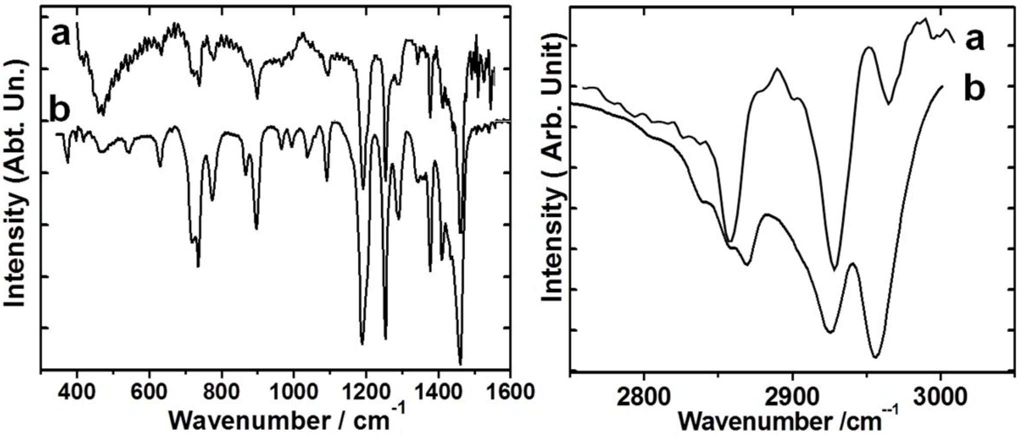

Figure SI.7:IR spectrum of dibutyl-diselenol. a) the dibutyl-diselenol dropped in silicon substrate; b) the dibutyl-diselenol linked in gold particles surface

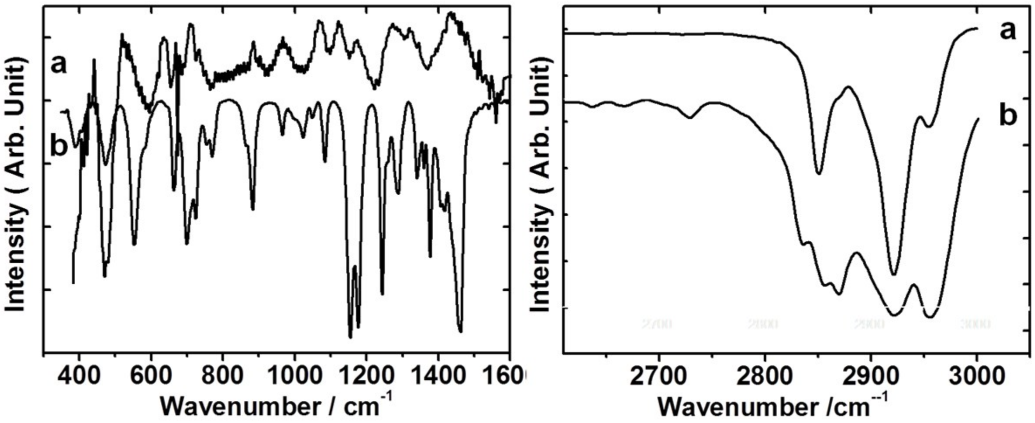

Figure SI.8: IR spectrum of dibutyl-ditellurol. a) the dibutyl-ditellurol dropped in silicon substrate; b) the dibutyl-ditellurol linked in gold particles surface

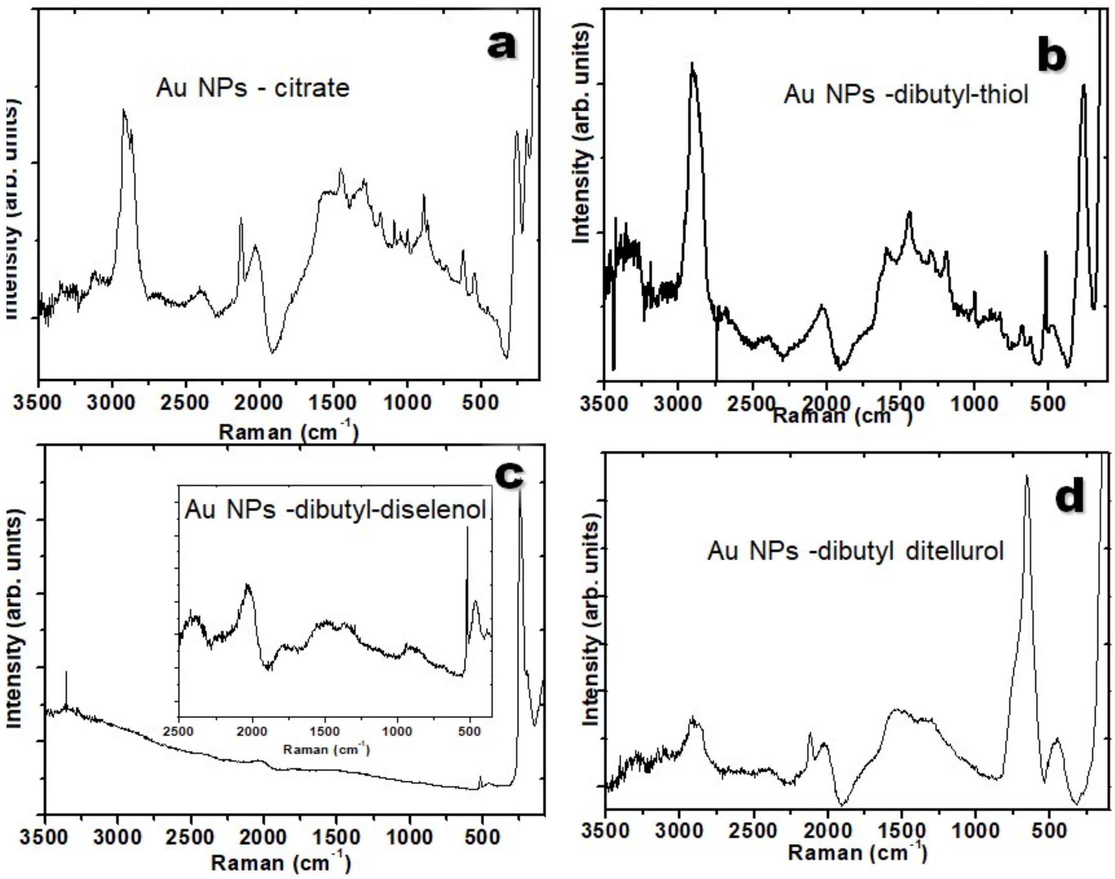

Figure SI.9: Raman spectrum of gold particles passivated with citrate and dibutyl-dichalcgenides. a) citrate; b)dibutyl-thiol S2d; c) dibutyl-disellenol Se3d; d) dibutyl-ditelurol. The Raman scattering of the sample obtained with 40 scans at 100mWat a resolution of 0.2cm-1 s-1 in the 100-3500 cm-1 region for solid-state sample in filme.

5. XPS ANALYSIS OF DIBUTYL-DICHALCOGENIDES MONOLAYES

In XPS measurements, the intensity of peaks provides quantitative information on the composition of the material surface, while the exact position of each peak yield information on the chemical state of the emitter atom. The bond type and number of electrons in the outer shell of the atom have direct influence on the binding energy of the electrons from the inner layers. When the electrons are removed or shared by an electronegative species, the effective charge felt by the inner electron is increased, thus resulting in an increase in binding energy resulting in the peak shifted to the higher energy region. XPS measurements revealed the existence of dibutyl-dichalcogenides species on the surface, for all samples peaks of Au and C were observed.

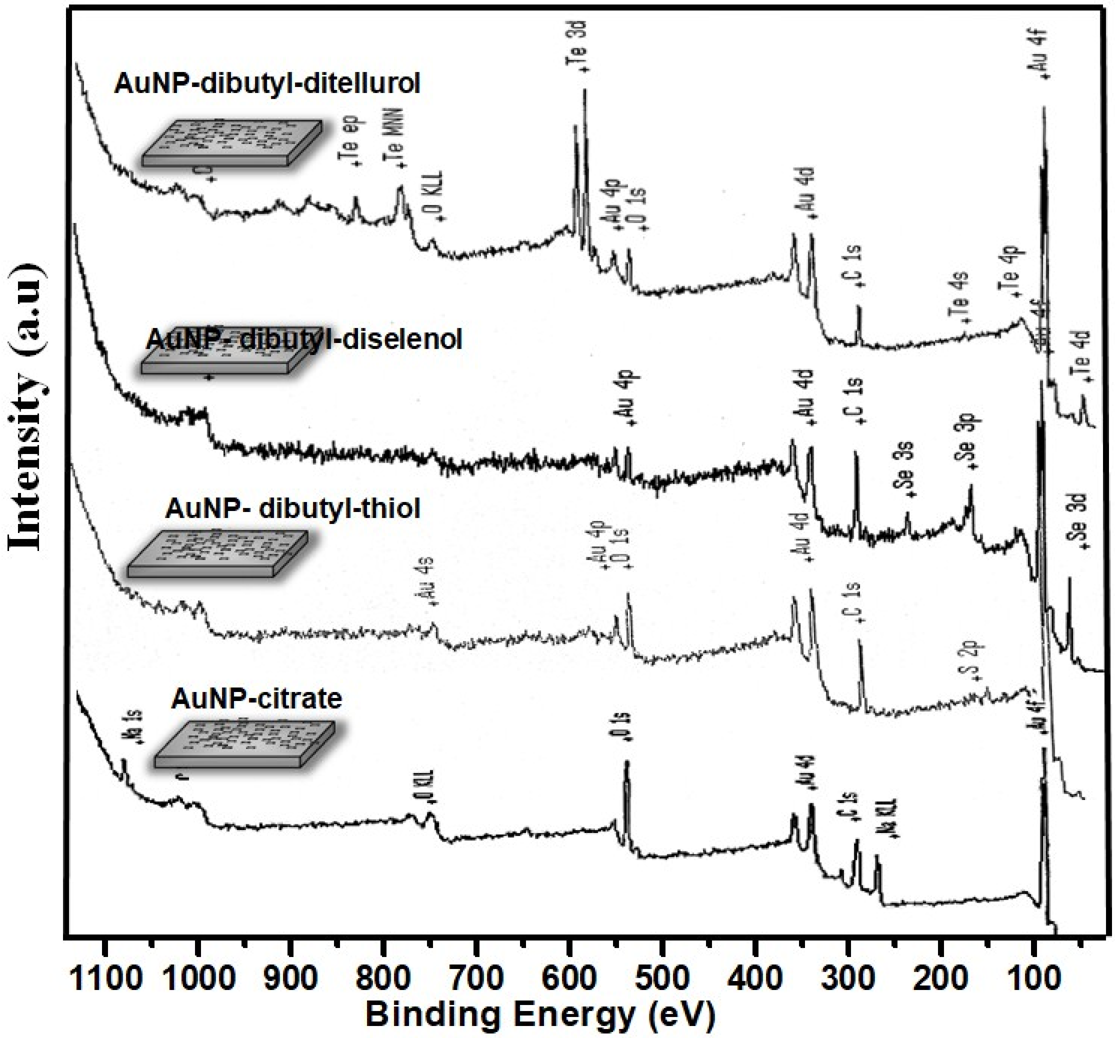

Figure SI.10: XPS spectrum of gold particles passivated with citrate and Au-dibutyl-dichacogenides,

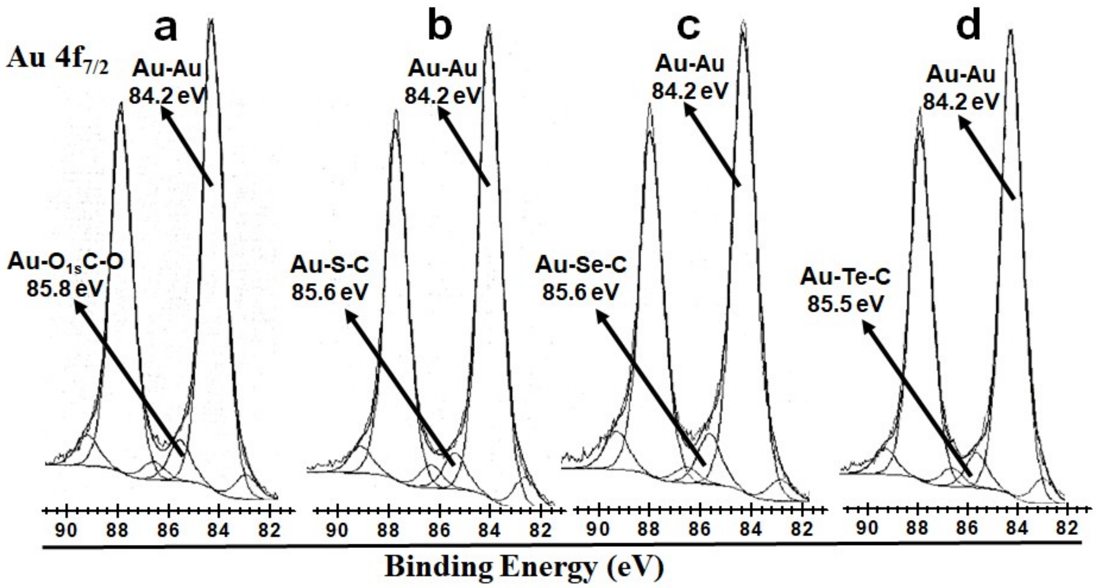

Figure SI-11**:** XPS spectrum of gold particles with surface passivated a) citrate Au $4f_{7/2}$ b) dibutyl-thiol Au $4f_{7/2}$; c) dibutyl-diselenol Au $4f_{7/2}$; d) dibutyl-ditellurol Au $4f_{7/2}$.

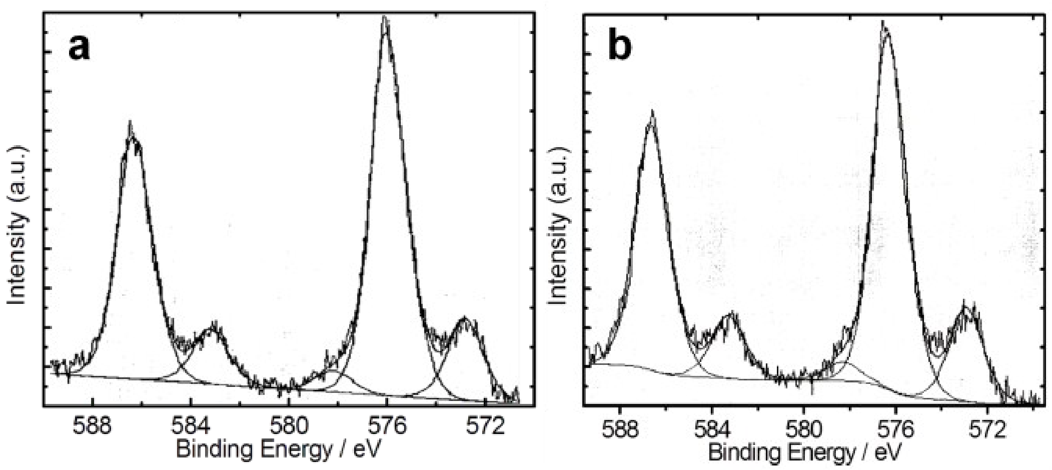

Figure SI.12: XPS spectrum of gold particles passivated with Au-dibutyl-ditellurol Te3d deposited in silicon substrate a) one days after synthesis; b) 13 months after synthesis.

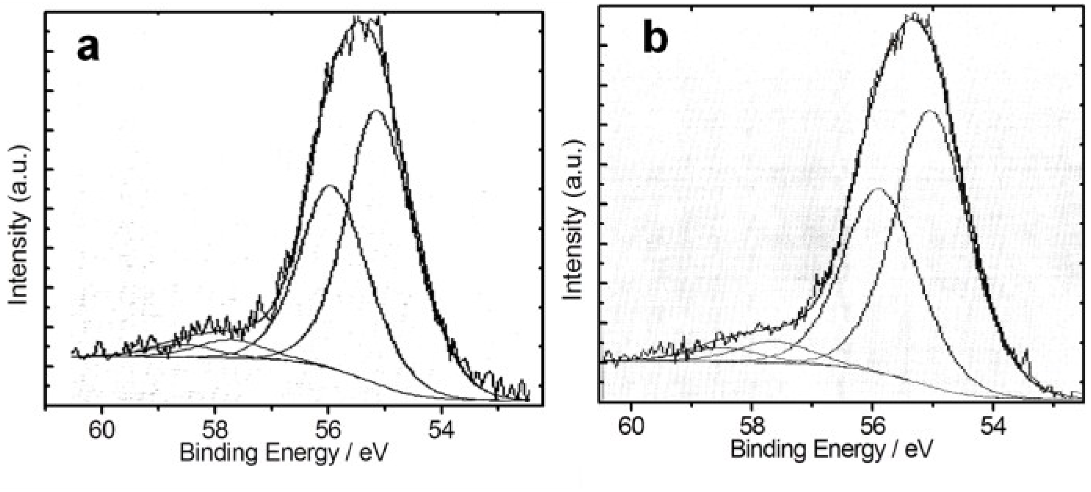

Figure SI.13: XPS spectrum of gold particles passivated with Au-dibutyl-diselenol Se3d deposited in silicon substrate. a) one days after synthesis; b) 13 months after synthesis.

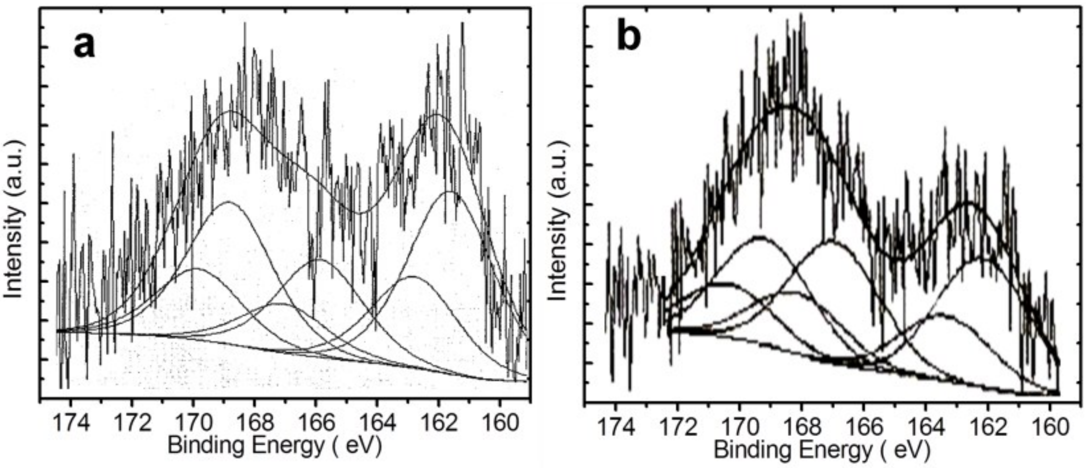

Figure SI.14: XPS spectrum of gold particles passivated with Au-dibutyl-thiol S2d deposited in silicon substrate. a) one days after synthesis; b) 13 months after synthesis.

Table SI-1. Values of the binding energies (in eV) for the of dibutyl-dichalcogenides molecules, values of oxidized molecules percentage one day after reaction

	Energia de Ligação (eV)		
	Te-Au	Se-Au	S-Au
C 1s	284,8	284,8 (77 %)<br>286,7 (18 %)<br>288,8 (5 %)	284,8 (71 %)<br>286,1 (23 %)<br>288,0 (6 %)
O 1s	530,3 (83 %)<br>532,1 (17 %)	531,5 (40 %)<br>533,1 (60 %)	532,1 (37 %)<br>533,1 (63 %)
Au $4f_{7/2}$	84,0 (93 %)<br>85,8 (7 %)	84,2 (90 %)<br>85,9 (10 %)	84,2 (93 %)<br>85,9 (7 %)
Te $3d_{5/2}$	572,9 (18 %)<br>575,9 (82 %)	—	—
Se $3d_{5/2}$	—	55,0 (94 %)<br>57,8 (6 %)	—
S $2p_{3/2}$	—	—	162,1 (42 %)<br>166,3 (23 %)<br>169,2 (35 %)

Table SI-2: Stability of dibutyl-dichalcogenides molecules in oxidation processes by oxygen species in the air under ambient conditions, values of oxidized molecules percentage one day and 13 months after reaction.

**Organic chalcogenides**	**oxidized molecules percentage ButX=O (X=S, Se and Te)**	
	**one day after reaction**	**13 months after reaction**
**dibuthyl-dithiol**	45%	49%
**dibuthyl-diselenol**	6%	7%
**dibuthyl-ditellurol**	82%	83%

Table SI-3. Compositions (% atomic) for the of dibutyl-dichalcogenides molecules, values of oxidized molecules percentage one day after reaction

	Composição (eV)		
	Te-Au	Se-Au	S-Au
C	43,54	60,29	56,80
O	19,36	11,02	20,69
Au	25,39	11,39	16,15
Te	11,71	—	—
Se	—	17,31	—
S	—	—	6,35

Table SI-4. Values of the binding energies (in eV) for the of dibutyl-dichalcogenides molecules, values of oxidized molecules percentage 13 month after reaction

	Energia de Ligação (eV)			
	AuS	AuSe	AuTe	AuC
C 1s	284,8 (76 %)<br>286,1 (20 %)<br>287,4 (4 %)	284,8 (84 %)<br>286,5 (13 %)<br>287,9 (3 %)	284,8 (74 %)<br>286,4 (19 %)<br>288,2 (7 %)	284,5 (8 %)<br>285,7 (41 %)<br>287,4 (27 %)<br>289,2 (18 %)<br>291,3 (5 %)
O 1s	531,8 (21 %)<br>533,0 (74 %)<br>534,9 (5 %)	531,7 (29 %)<br>533,1 (71 %)	530,4 (36 %)<br>532,1 (24 %)<br>533,1 (40 %)	532,2 (49 %)<br>533,7 (51 %)
Au $4f_{7/2}$	82,8 (6 %)<br>84,2 (87 %)<br>85,6 (7 %)	82,9 (4 %)<br>84,2 (86 %)<br>85,6 (10 %)	83,0 (5 %)<br>84,2 % (82 %)<br>85,7 (7 %)	83,0 (5 %)<br>84,2 (88 %)<br>85,6 (7 %)
S $2p_{3/2}$	162,3 (36 %)<br>167,0 (35 %)<br>169,3 (29 %)	—	—	—
Se $3d_{5/2}$	—	55,1 (93 %)<br>57,7 (7 %)	—	—
Te $3d_{5/2}$	—	—	572,8 (21 %)<br>576,1 (79 %)	—

Table SI-5. Compositions (% atomic) for the of dibutyl-dichalcogenides molecules, values of oxidized molecules percentage 13 month after reaction

	Composição (eV)			
	AuS	AuSe	AuTe	AuC
C	52,42	58,44	40,92	57,94
O	26,50	12,46	23,61	27,63
Au	14,68	11,45	29,83	11,14
S	6,39	—	—	—
Se	—	17,79	—	—
Te	—	—	5,65	—
Na	—	—	—	3,29

6. COMPUTATIONAL DATA ON THE OXIDATION ENERGIES OF THE CAPPED AU NPS

Table SI-6. Energies of all the species considered calculated at the PBEPBE/Lanl2dz level of theory.

E_0, A.U./E_0+ZPE, A.U.	
Au_{20}, C_1 ^1A	
-2710.009606/-2709.999633	
$X_2C_8H_{18}$, X = S, Se, Te	
$S_2C_8H_{18}$, C_1 ^1A	-335.404472/-335.160372 (cis)
$S_2C_8H_{18}$, C_1 ^3A	-335.360035/-335.117349 (cis)
$S_2C_8H_{18}$, C_1 ^1A	-335.404470/-335.160389 (trans)
$S_2C_8H_{18}$, C_1 ^3A	-335.363246/-335.120200 (trans)
$Se_2C_8H_{18}$, C_1 ^1A	-333.666265/-333.423940 (cis)
$Se_2C_8H_{18}$, C_1 ^3A	-333.621853/-333.380460 (cis)
$Te_2C_8H_{18}$, C_1 ^1A	-331.332428/-331.091294 (trans)
$Te_2C_8H_{18}$, C_1 ^1A	-331.332431/-331.091354 (cis)
$Au_{20}(X_2C_8H_{18})$, X = S, Se, Te//ΔE, kcal/mol//BE(ligand), kcal/mol	
$Au_{20}(S_2C_8H_{18})$, C_1 ^1A	-3045.433825/-3045.179646//0.0//12.4/12.3
$Au_{20}(S_2C_8H_{18})$, C_1 ^3A	-3045.420893/-3045.166889//8.1/8.0
$Au_{20}(Se_2C_8H_{18})$, C_1 ^1A	-3043.701909/-3043.449040//0.0/16.3/16.0
$Au_{20}(Se_2C_8H_{18})$, C_1 ^3A	-3043.693361/-3043.441343//5.4/4.8//
$Au_{20}(Te_2C_8H_{18})$, C_1 ^1A	-3041.372118/-3041.121222//6.1/6.0
$Au_{20}(Te_2C_8H_{18})$, C_1 ^3A	-3041.381839/-3041.130833//25.0/
$Au_{20}(X_2C_8H_{18})_2$, X = S, Se, Te // BE, kcal/mol	
$Au_{20}(S_2C_8H_{18})_2$, C_1 ^1A	-3380.863019/-3380.364137//27.9/27.5
$Au_{20}(Se_2C_8H_{18})_2$, C_1 ^1A	-3377.394412/-3376.899024//32.8/32.3
$Au_{20}(Se_2C_8H_{18})_2$, C_1 ^3A	-3377.386233/-3376.891229
$Au_{20}(Te_2C_8H_{18})_2$, C_1 ^1A	-3372.743341/-3372.250498//21.6/
$Au_{20}(Te_2C_8H_{18})_2$, C_1 ^3A	-3372.739691/-3372.247541
$Au_{20}(X_2C_8H_{18})_2O_2$, X = S, Se, Te	
$Au_{20}(S_2C_8H_{18})_2O_2$, C_1 ^3A	-3195.638893/-3195.380216
$Au_{20}(Se_2C_8H_{18})_2O_2$, C_1 ^3A	-3193.913508/-3193.656986
$Au_{20}(Te_2C_8H_{18})_2O_2$, C_1 ^3A	-3191.602851/-3191.347465
$Au_{20}(X_2O_2C_8H_{18})_2$, X = S, Se, Te	
$Au_{20}(S_2O_2C_8H_{18})_2$, C_1 ^3A	-3681.221062/-3680.713813
$Au_{20}(Se_2O_2C_8H_{18})_2$, C_1 ^3A	-3677.795342/ -3677.291450
$Au_{20}(Te_2O_2C_8H_{18})_2$, C_1 ^3A	-3673.177224/-3672.676527
O_2, ^3A	-150.17788
$2O_2$	-300.35576
$\Delta E(Au_{20}(X_2C_8H_{18})_2 + 2O_2 \Rightarrow Au_{20}(X_2O_2C_8H_{18})_2)$, X = S, Se, Te, kcal/mol / eV	
S	-1.43/-0.062
Se	-33.48/-1.45
Te	-51.31/-2.23
$E(Au_{20}(X_2C_8H_{18})_2 + O_2 \Rightarrow Au_{20}(X_2C_8H_{18})_2O_2)$, X = S, Se, Te, kcal/mol / eV	
S	-17.1/-0.74
Se	-21.1/-0.91
Te	-26.2/-1.14

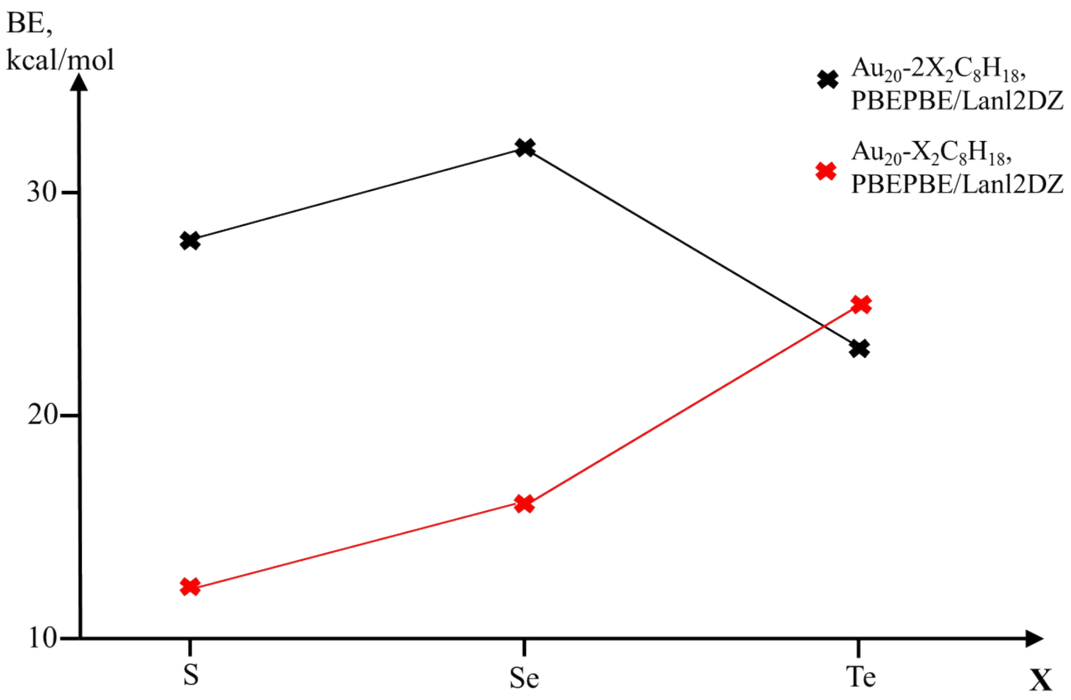

Figure SI.15: Plots of binding energies, kcal/mol, at the PBEPBE/Lanl2dz level, for the Au_{20} NP capped with 1 (red) and 2 (black) $X_2C_8H_{18}$ ligands.

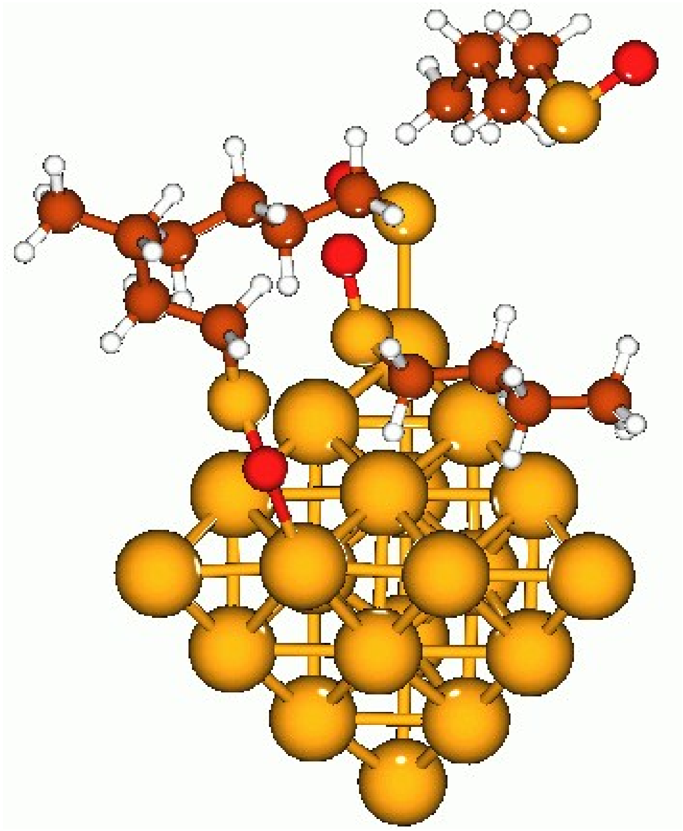

Figure SI.16: Optimized triplet structure of the Au_{20} NP capped with 2 $S_2C_8H_{18}$ ligands and oxidized by 2 O_2 molecules, at the PBEPBE/Lanl2dz level (large golden balls stand for Au, small golden balls denote S, dark brown balls denote C, red balls denote O, and white balls denote H).

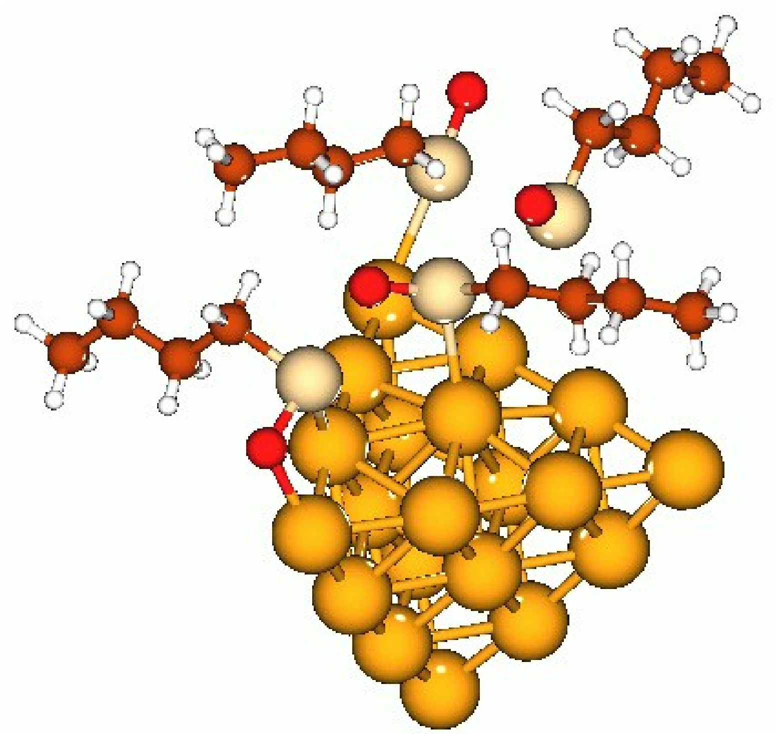

Figure SI.17: Optimized triplet structure of the Au_{20} NP capped with 2 $Se_2C_8H_{18}$ ligands and oxidized by 2 O_2 molecules, at the PBEPBE/Lanl2dz level (large golden balls stand for Au, light brown balls denote Se, dark brown balls denote C, red balls denote O, and white balls denote H).

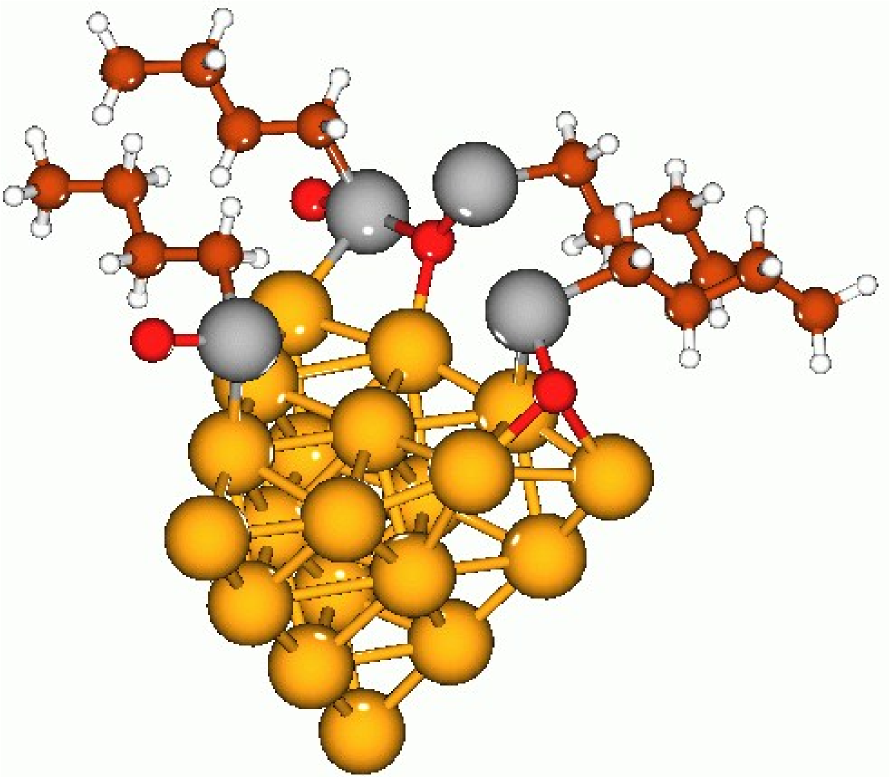

Figure SI.16: Optimized triplet structure of the Au_{20} NP capped with 2 $Te_2C_8H_{18}$ ligands and oxidized by 2 O_2 molecules, at the PBEPBE/Lanl2dz level (large golden balls stand for Au, grey balls denote Te, dark brown balls denote C, red balls denote O, and white balls denote H).

Discussion

Gold nanoclusters/nanoparticles (Au NCs or Au NPs) in the quantum size regime have attracted considerable research interest in last decades due to their promising optical, chiral, magnetic and catalytic properties [1,2]. Their sizes are comparable to the de Broglie wavelength of electrons near the Fermi energy of metallic gold, thus the few-atom Au NCs can show molecule-like interactions with incident light to produce intense emission [3-5]. Au NCs have such useful properties as broad excitation ranges, size-dependent emission color, red/near-infrared (NIR) emission and diminished toxicity, which makes them ideal for applications in cellular and subcellular imaging [6]. Significant efforts have been devoted to the design of biocompatible gold clusters [see, e.g., Ref. 7].

Understanding the surface behavior of various ligands on the Au NC surface and their effects on structures and properties of Au NCs is highly important and challenging task. The most stable and well characterized atomically precise gold−thiolate (SR) nanocluster is $Au_{25}(SR)_{18}$, whose structure and optical properties have been a subject of intense research [see, e.g., Ref. 8]. From an electronic perspective, the $Au_{25}(SR)_{18}$ species is often considered as a “superatom” [9], with frontier orbitals almost exclusively contributed by the 13 Au atoms of its icosahedral kernel [10]. The core−shell geometric and electronic structures are a prominent feature of many Au NCs [11]. In addition to numerous experimental studies, versatile theoretical approaches have been shown to be able to provide insights into the electronic, structural and dynamic behavior of the ligand-capped Au NCs [12]. Thus, time-dependent density functional theory (TDDFT) studies of the $[Au_{25}(SR)_{18}]^-$ NC (R = H, CH_3, CH_2CH_3, $CH_2CH_2CH_3$) before and after photoexcitation revealed that all excited states arose from core-based orbitals, which indicated that ligands primarily affect photoluminescence via their interactions with the NC core [13]. It was shown that structural relaxations in the optimized excited states of up to 0.33 Å imparted remarkable effects on the energy levels of the frontier orbitals of the nanoclusters studied. Also, DFT studies showed that electron-withdrawing ligands can distort the $Au_{25}S_{18}$ framework, and that these structural changes were strongly correlated to a reduction of the HOMO−LUMO gap [14]. Also, the quantum mechanics/molecular mechanics (QM/MM) approach was employed to demonstrate that the HOMO−LUMO gap of the $[Au_{25}(SG)_{18}]^-$ (SG = glutathione) and $[Au_{25}(SCH_3)_{18}]^-$ depended sensitively on both the ligands and the solvent [15].

In 2018, Lin et al. demonstrated that the emission intensity of novel oligopeptide-protected Au NCs was closely related to the local chemical environment as manifested via the peptide layer structuring and dynamics near the gold surface [11]. It was shown that the peptide sequence plays an important role in determining the surface peptide structuring, interfacial water dynamics and ligand-Au core interactions, which can be tailored by controlling peptide acetylation, constituent amino acid electron donating/ withdrawing capacity, aromaticity/hydrophobicity and by adjusting environmental pH. These findings provided key design principles for understanding the surface dynamics of peptide-protected Au NPs and maximizing the photoluminescence of metallic clusters through the exploitation of biologically relevant ligand properties. *Molecular dynamics (MD) simulations* were employed in this research. The Au NC structure used in the MD simulations contained 25 Au atoms and was morphology-consistent with the highly stable $Au_{25}(SR)_{18}$ NCs, which can conceptually be divided into an icosahedral Au_{13}-core protected by six –[SR–(Au-SR)$_2$]– "staple" motifs. The peptide-coated Au NCs models were constructed by attaching, via the N-terminal cysteine, 18 extended peptide ligands equidistantly onto the X-ray crystal structure of $Au_{25}(SR)_{18}$. All peptides were NH_2-capped on the C-terminus and N-terminated with either a NH_3^+ moiety or a CH_3CO-group (denoted by an "Ac-" prefix). In total, 14 different $Au_{25}(SP)_{18}$ (P = peptide) simulation models were constructed with the following peptide sequences: CGGGDD (control); CVGGDD, CHGGDD, CYGGDD, CGYGDD, CHYGDD, CYHGDD, CYYGDD, and CVVGDD to explore hydrophobicity and individual amino acid location; Ac-CGGGDD, Ac-CHGGDD, Ac-CHYGDD and Ac CYHGDD to study N-terminal acetyl capping; along with an additional positively charged peptide with arginine residues, CYYGRR, to investigate a cellular internalizing sequence. The chosen models represented diverse experimental systems. Explicit solvent MD simulations were conducted using the GROMACS 4.6.5 software. Peptide interatomic interactions were modelled using the all-atom AMBER99SB-ILDN force field (FF). The TIP3P model was used for water. Bonded parameters and Lennard-Jones potentials between Au and S, C, and H were adopted from a FF parameterized for similar monolayer-protected Au NCs assuming no explicit partial charges on Au. Additional parameters for the Au-S-C-C dihedrals were obtained from dihedral scan at the B3LYP/6-31G* level (LanL2DZ basis set for Au) in Gaussian09. For non-bonded interactions, long-range electrostatics were evaluated using the Particle Mesh Ewald (PME) method with areal space cutoff of 10 Å and a 1.2 Å fast Fourier transform (FFT) grid spacing, while van der Waals interactions

were truncated at 10 Å. To preserve the core crystal structure of $Au_{25}(SP)_{18}$ during MD, distance restraints of 1000 kJ/mol/nm^2 were applied between Au-Au atoms. Each $Au_{25}(SP)_{18}$ model was placed in a periodic cubic box with side length ~8.4 nm, solvated with ~19000 water molecules (water density of ~1 g/cm3) and Na^+ or Cl^- counter ions were added to ensure a neutral simulation cell. Energy minimization was carried out using the steepest descent algorithm to remove any steric clashes and 1 ns of position restrained MD was performed using the Berendsen thermostat and barostat to equilibrate the solvent around the Au NCs at 300 K temperature and 1 atm pressure. Position restraints were then removed and 100 ns of NPT (constant pressure and temperature ensemble) MD was performed with the Nosé-Hoover thermostat and Parrinello-Rahman barostat to maintain temperature and pressure at 300 K and 1 atm. The LINCS algorithm was applied to constrain all bonds to their equilibrium lengths, which enabled a time-step of 2 fs to be used for each simulation. Frames were outputted to a trajectory file every 2 ps. To enhance conformational sampling, each system was simulated 10 times starting from different initial atomic velocities, resulting in a total of 1 μs of data for each $Au_{25}(SP)_{18}$. The properties and structures presented in the paper were ensemble averaged over the 10 independent trajectories for each $Au_{25}(SP)_{18}$ system and, unless stated otherwise, analysis was performed on the thermally equilibrated stage of the simulations covering the final 20 ns of each trajectory (200 ns of production data per system). Quantum mechanics (QM) calculations were performed in the following way: initial geometries of the $Au_{25}S_{18}$Cys Au NCs with acetyl, protonated amine, and deprotonated amine N-termini were taken from equilibrated MD simulation snapshots. Geometry optimization of the cysteine ligands with N-methylamine C-termini was performed using the DMoL3 program with the PBE functional and double-numeric polarized (DNP) basis se with all-electron relativistic core treatment. Solvent effects were treated using the conductor-like screening model S4(COSMO). Electrostatic potential (ESP) atomic partial charge calculations of the optimized structures were performed using Gaussian09, with the PBE functional and the relativistic double-ζ LanL2DZ basis set and effective core potential (ECP) for Au atoms, and the 6-311G(d,p) basis set for other atoms. Solvent effects were treated using the Polarizable Continuum Model (PCM).

As can be seen, the understanding of the structures and properties of ligand-capped Au NCs/NPs in aqueous and biological environments can be gained by employing atomistic molecular simulations which can offer a detailed view. Quite a few other MD simulation studies have been reported in the last decades devoted to the investigation of

the following issues. (i) The Au-S bond nature [12, 16, 17]. In the 2012 review of Häkkinen [16] mostly DFT investigations of thiolate-protected Au NCs are described, including the $Au_{38}(SR)_{24}$ NC, and in the 2014 study by Brancolini et al. [17] a computational model of the effect of a prototypical thiol-protected gold NP, $Au_{25}L_{18}^{-}$ (L = $S(CH_2)_2Ph$) on the □$_2$-microglobulin natural fibrillation propensity is provided. To reveal the molecular basis of the protein–NP association process, various simulations at multiple levels (Classical MD and Brownian Dynamics) were performed that covered multiple length- and timescales. The results provided a model of the ensemble of structures constituting the protein–gold NP complexes along with insights into the driving forces for the binding of □$_2$-microglobulin to hydrophobic small size gold NPs. Is was found that the small nanoparticles can bind the protein to form persistent complexes and this binding of nanoparticles was able to block the active sites of domains from binding to another protein, thus leading to potential inhibition of the fibrillation activity. (ii) The solvent structuration at the interface with naked or ligand-protected Au NPs in aqueous and organic solvents and the conformation of the protecting ligand chains [18–26]. Thus, Lane and Grest in 2010 investigated the symmetry, and the deviation from it, of decane-octadecanethiol ligand chains on a Au NP at liquid-vapor interface [19]. In a more recent work, Bolintineanu et al. reported classical atomistic MD simulations of alkanethiol-coated gold nanoparticles with diameters of 4 nm solvated in water and decane, as well as at water/vapor interfaces. The ligand molecules investigated were $HS(CH_2)_9X$ and $HS(CH_2)_{17}X$, with X being one of the following end groups: CH_3, COOH, $COO^{-}Na^{+}$, $COO^{-}Ca^{2+}$, NH_2, and $NH_3^{+}Cl^{-}$. In all cases, coatings were fully homogeneous with respect to ionization state and alkane chain length. In addition, the uncharged cases (CH_3, COOH, and NH_2) were simulated in bulk decane to investigate the effects of solvent interactions. Significant coating asymmetry was observed, particularly for nanoparticles coated with $S(CH_2)_{17}X$ ligands solvated in water. The asymmetry was attenuated by charged end groups (X = $COO^{-}Na^{+}$, $COO^{-}Ca^{2+}$, or $NH_3^{+}Cl^{-}$), consistent with the propensity of charged $S(CH_2)_9X$ particles to enter the water phase in water-vapor simulations. Decane-solvated particles also showed some coating asymmetry, which was largely due to crystallization of the ligands. On the basis of the coating structures and density profiles, it was possible to qualitatively infer the overall solubility of the NPs. This asymmetry in the alkanethiol coatings was likely to have a significant effect on aggregation behavior. The simulations elucidated the mechanism by which modulating

the end group charge state can be used to control coating structure and therefore nanoparticle solubility and aggregation behavior.

Yang and Weng in 2010 reported a detailed study on structural and dynamic properties of water interface close to a non-charged, polar and nonpolar Au NPs functionalized with different terminal groups of the capping chains [25]. In 2012, Heikkilä et al. [20] reported the study of charged monolayer-protected Au NPs in aqueous solution by performing atomistic MD simulations at physiological temperature (310 K). Particular attention was paid to electrostatic properties that modulate the formation of a complex comprised of the NP together with surrounding ions and water. The focus was on the Au_{144} nanoparticles that comprised a nearly spherical Au core (diameter ∼2 nm), a passivating Au–S interface, and functionalized alkanethiol chains. Cationic and anionic Au NPs were modeled with amine and carboxyl terminal groups and Cl^{-}/Na^{+} counterions, respectively. The radial distribution functions showed that the side chains and terminal groups had significant flexibility. The orientation of water was distinct in the first solvation shell, and Au NPs caused a long-range effect in the solvent structure. The radial electrostatic potential displayed a minimum for Au NP^{-} at 1.9 nm from the center of the nanoparticle, marking a preferable location for Na^{+}, while the Au NP^{+} potential (affecting the distribution of Cl^{-}) was found to rise almost monotonically with a local maximum. The results highlighted the importance of long-range electrostatic interactions in determining nanoparticle properties in aqueous solutions. They suggested that electrostatics was one of the central factors in complexation of Au NPs with other nanomaterials and biological systems, and that effects of electrostatics as water-mediated interactions were relatively long-ranged, which likely plays a role in, e.g., the interplay between nanoparticles and lipid membranes that surround cells. In 2015, Yang et al. performed a MD study to investigate the structure and dynamic properties of hydrogen bond (HB) and infrared spectra of hydration water around monolayer-protected Au NPs [27]. Also in 2015, Giri and Spohr performed atomistic MD simulations of ligand-capped Au_{144} nanoparticles in aqueous NaCl solution [21]. Alkanethiol chain-covered NPs at grafting densities between approximately one-third and full coverage were studied with nonpolar CH_3 and charged COO^{-} and NH_3^{+} terminations. Special attention was given to the penetration depth of water and ions into the diffuse shell of the functionalized alkanethiol chains and its dependence on grafting density and functionalization. High grafting densities were found to lead to more extended hydrocarbon chains. Charged functionalized Au NPs were found to produce non-monotonous counter charge

distributions with reduced ion mobility. Partial replacement of first shell solvation water by the charged groups was found to lead to a drastic increase in torsional relaxation times of the chain termini. Due to the large curvature of the Au NPs with a diameter of 2 nm, gold cores remained accessible to both ions and water even at the highest studied grafting densities of about 5 chains/nm^2. In the recent MD study by these authors on the ligand-capped Au_{144} NPs the Y-shape of the terminal groups was considered [22]. Also in 2018, Chew and Lehn [23] and Kister et al. [24] reported detailed MD studies to understand the colloidal stability and the role of the Au core morphology on the bundle formation and asymmetry of grafted chains of the Au NPs. In the first case, the Au NPs core sizes were 2, 3, 4, 5, 6, and 7 nm [23], and in the second study 7 different mean core diameters between 4 nm and 9.7 nm were studied [24]. Very recently, Simonelli et al. addressed, by MD at coarse-grained resolution, the interactions between human serum albumin (HSA) and a prototypical monolayer-protected Au nanoparticle [26]. The Au NP core had a small diameter of 2 nm. The Au NP surface was covalently functionalized by ligands terminated by a zwitter-ionic group and, at the same time, having tunable hydrophobicity. The authors showed that both the chemical composition (charge, hydrophobicity) and the conformational preferences of the ligands decorating the NP surface affected the NP propensity to bind HSA.

Also in 2019, Yamanaka et al. performed atomistic MD simulations, to investigate the ligand-capped Au NP/water interface in pure and salt aqueous solutions [27]. The authors selected two different Au NP coatings: (i) grafted chains carrying different zwitter-ionic sulfobetaine groups, which were shown to reduce/avoid the protein corona formation on NPs in physiological conditions and, (ii) noncharged pegylated ligands. By using an atomistic model, based on the OPLS-AA force-field, first comparison of the hydrodynamic sizes of NPs was performed, finding similar values between calculated and experimental data set. The authors observed changes in the NP shape from an asymmetric and elongated shape (i.e. more compact) to a spherical one with less packed chains resembling a fully-blossomed flower, as function of the R substituent on the amino group of the zwitterionic sulfobetaine head. They designated this shape change by chain blossom. For less bulky R substituents the formation of discrete bundles in which the ligand chains were well packed was observed, while more uniform and quasispherical distribution of ligands onto Au core was observed for the bulkiest R group consistently with previous studies for similar ligand chains. Furthermore, the increase of the length of R alkyl groups was shown to cause an increase of bulkiness and hydrophobicity with both

properties modulating the shape of NPs. At molecular level, it was shown that the chain-blossom change was guided by the free volume/ligand chain. In particular, when the chains were grouped in bundles, the free volume/chain was larger than chain volume which implied that chains had more available hydrophobic volume exposed to the solvent. In this case, the bundle shape was shown to minimize
the hydrophobic volume in contact with the solvent. For the spherical case where the free volume/chain was lower, the scenario was the opposite. Therefore, the characterization of NP shape and NP/water interface was reported from the analyses of pair distance distribution, eccentricity parameter, atom distributions in spherical coordinates and RDF. In addition, the interaction of NPs' surface with its aqueous surrounding was investigated by computing the H-Bond fraction, SASA and the Electrostatic Surface Potential. It was found that TEG shows the lower SASA value but the highest capability to form H-bonds. On the other hand, the NPs containing sulfobetaine as terminal groups were found to exhibit larger SASA value but the increase of the R bulkiness corresponded to an increase in the screening of the polar groups to the water exposure, reducing the whole capability to form H-bond. The emerging picture from the MD simulations demonstrated that the NP size was not the only factor influencing NP interaction with the water solvent molecules, but the shape and the local organization of the ligand chains played a strong role in that. This behavior should be considered as an indicator of the capability of the NPs to interact with hydrophilic protein sites.

Also, it is worthwhile to mention the following DFT studies. (i) The 2016 study by Weerawardene and Aikens, where the authors performed a theoretical investigation using DFT and TDDFT [29]. They optimized a methylthiolate version of the new crystal structure geometry of $Au_{20}(TBBT)_{16}$ and compared the stability and optical properties with the three lowest energy isomers of $Au_{20}(SCH_3)_{16}$ predicted previously. Furthermore, TDDFT calculations were performed for the $Au_{20}(TBBT)_{16}$ structure determined via X-ray crystallography and the related $Au_{20}(SPh)_{16}$ nanocluster. (ii) 2012 DFT calculations performed by Jung et al. which suggested that Au_m cluster structures were insensitive to ligands R for all the thiolate-stabilized Au_{25}, Au_{38}, and Au_{102} clusters. (iii) 2018 work by Kenzler et al. which showed that reducing $(Ph_3P)AuSC(SiMe_3)_3$ with L-Selectride gave the medium-sized metalloid gold cluster $Au_{70}S_{20}(PPh_3)_{12}$ [31]. Their DFT studies showed that the phosphine bound Au-atoms not only stabilized the electronic structure of $Au_{70}S_{20}(PPh_3)_{12}$, but also behaved as electron acceptors leading to auride-like gold atoms on the exterior.

Thus, we can conclude from the above-provided review of DFT and MD studies of various ligand-capped Au NPs of different sizes, including both smaller and bigger sizes, up to ca. 10 nm: *there have been no computational studies reported* for oxygen interaction/oxidation or stability towards oxidation of Au NPs capped with S-containing ligands, as well as no similar studies for Au NPs capped with Se/Te-containing ligands. Also, it is necessary to emphasize that even in MD simulations smaller models for Au NPs have been employed (see above). Thus, using our model Au_{20} NP would be justified as a first step for the studies for oxygen interaction/oxidation or stability towards oxidation of Au NPs capped with S/Se/Te-containing ligands. Also, this model was employed because the calculations with bigger models would be quite time- and resource-consuming.

References

1. Qian, H.; Zhu, M.; Wu, Z.; Jin, R. Acc. Chem. Res. 2012, 45, 1470−1479.
2. Jin, R.; Zeng, C.; Zhou, M.; Chen, Y. Chem. Rev. 2016, 116, 10346−10413.
3. Bigioni, T. P.; Whetten, R. L.; Dag, Ö. J. Phys. Chem. B 2000, 104, 6983−6986.
4. Zheng, J.; Petty, J. T.; Dickson, R. M. J. Am. Chem. Soc. 2003, 125, 7780−7781.
5. Link, S.; Beeby, A.; FitzGerald, S.; El-Sayed, M. A.; Schaaff, T. G.; Whetten, R. L. J. Phys. Chem. B 2002, 106, 3410−3415.
6. Du, B.; Jiang, X.; Das, A.; Zhou, Q.; Yu, M.; Jin, R.; Zheng, J. Nat. Nanotechnol. 2017, 12, 1096.
7. Goswami, N.; Zheng, K.; Xie, J. Nanoscale 2014, 6, 13328−13347.
8. Kang, X.; Chong, H.; Zhu, M. Nanoscale 2018, 10, 10758−10834.
9. da Silva, R. R.; Ramalho, T. C.; Santos, J. M.; Figueroa-Villar, J. D. J. Phys. Chem. A 2006, 110, 1031−1040.
10. Zhu, M.; Aikens, C. M.; Hollander, F. J.; Schatz, G. C.; Jin, R. J. Am. Chem. Soc. 2008, 130, 5883−5885.
11. Surface Dynamics and Ligand−Core Interactions of Quantum Sized Photoluminescent Gold Nanoclusters. Yiyang Lin, Patrick Charchar, Andrew J. Christofferson, Michael R. Thomas, Nevena Todorova, Manuel M. Mazo, Qu Chen, James Doutch, Robert Richardson, Irene Yarovsky, and Molly M. Stevens. J. Am. Chem. Soc. 2018, 140, 51, 18217-18226.
12. Charchar, P.; Christofferson, A. J.; Todorova, N.; Yarovsky, I. Understanding and Designing the Gold–Bio Interface: Insights from Simulations. Small 2016, 12, 2395−2418.
13. Weerawardene, K. L. D. M.; Aikens, C. M. J. Am. Chem. Soc. 2016, 138, 11202−11210.
14. Tlahuice-Flores, A.; Whetten, R. L.; Jose-Yacaman, M. J. Phys. Chem. C 2013, 117, 20867−20875.
15. Rojas-Cervellera, V.; Rovira, C.; Akola, J. J. Phys. Chem. Lett. 2015, 6, 3859−3865.
16. H. Häkkinen, The gold-sulfur interface at the nanoscale, Nat. Chem. 4 (2012) 443–455.
17. G. Brancolini, D. Toroz, S. Corni, Can small hydrophobic gold nanoparticles inhibit β_2-microglobulin fibrillation? Nanoscale 6 (2014) 7903–7911.
18. D.S. Bolintineanu, J.M.D. Lane, G.S. Grest, Effects of functional groups and ionization on the structure of alkanethiol-coated gold nanoparticles, Langmuir 30 (2014) 11075–11085.
19. J.M.D. Lane, G.S. Grest, Spontaneous asymmetry of coated spherical nanoparticles in solution and at liquid-vapor interfaces. Phys. Rev. Lett. 104 (2010) 9–12.
20. E. Heikkilä, A.A. Gurtovenko, H. Martinez-Seara, H. Häkkinen, I. Vattulainen, J. Akola, Atomistic simulations of functional $Au_{144}(SR)_{60}$ gold nanoparticles in aqueous environment, J. Phys. Chem. C. 116 (2012) 9805–9811.

21. A.K. Giri, E. Spohr, Conformational equilibria of organic adsorbates on nanostructures in aqueous solution: MD simulations, J. Phys. Chem. C. 119 (2015) 25566–25575.
22. A.K. Giri, E. Spohr, Influence of chain length and branching on the structure of functionalized gold nanoparticles, J. Phys. Chem. C. 122 (2018) 26739–26747.
23. A.K. Chew, R.C. Van Lehn, Effect of core morphology on the structural asymmetry of alkanethiol monolayer-protected gold nanoparticles, J. Phys. Chem. C. 122 (2018) 26288–26297.
24. T. Kister, D. Monego, P. Mulvaney, A. Widmer-Cooper, T. Kraus, Colloidal stability of apolar nanoparticles: the role of particle size and ligand shell structure, ACS Nano 12 (2018) 5969–5977.
25. A.-C. Yang, C.-I. Weng, Structural and dynamic properties of water near monolayer-protected gold clusters with various alkanethiol tail groups, J. Phys. Chem. C. 114 (2010) 8697–8709.
26. F. Simonelli, G. Rossi, L. Monticelli, Role of ligand conformation on nanoparticle-protein interactions, J. Phys. Chem. B. 123 (2019) 1764–1769.
27. Z. Yang, Y. Li, G. Zhou, X. Chen, D. Tao, N. Hu, Molecular dynamics simulations of hydrogen bond dynamics and far-infrared spectra of hydration water molecules around the mixed monolayer-protected Au nanoparticle, J. Phys. Chem. C. 119 (2015) 1768–1781.
28. Tsudo Yamanaka, Antonio De Nicola, Gianmarco Munaò, Thereza A. Soares, Giuseppe Milano. Effect of the ligand's bulkiness on the shape of functionalized gold nanoparticles in aqueous solutions: A molecular dynamics study. Chemical Physics Letters 731 (2019) 136576.
29. K. L. Dimuthu M. Weerawardene and Christine M. Aikens. Effect of Aliphatic versus Aromatic Ligands on the Structure and Optical Absorption of $Au_{20}(SR)_{16}$. J. Phys. Chem. C 2016, 120, 15, 8354-8363.
30. Jung, J.; Kang, S.; Han, Y.-K., Ligand Effects on the Stability of Thiol-Stabilized Gold Nanoclusters: $Au_{25}(SR)_{18}^{-}$, $Au_{38}(SR)_{24}$, and $Au_{102}(SR)_{44}$. Nanoscale 2012, 4, 4206-4210.
31. Sebastian Kenzler, Claudio Schrenk, Andrew R. Frojd, Hannu Häkkinen, Andre Z. Clayborne and Andreas Schnepf. $Au_{70}S_{20}(PPh_3)_{12}$: an intermediate sized metalloid gold cluster stabilized by the Au_4S_4 ring motif and Au-PPh_3 groups. Chem. Commun., 2018, 54, 248.
32. Yongbo Song, Shuxin Wang, Jun Zhang, Xi Kang, Shuang Chen, Peng Li, Hongting Sheng, and Manzhou Zhu. Crystal Structure of Selenolate-Protected $Au_{24}(SeR)_{20}$ Nanocluster. J. Am. Chem. Soc. 2014, 136, 2963−2965.
33. Wataru Kurashige, Seiji Yamazoe, Masaki Yamaguchi, Keisuke Nishido, Katsuyuki Nobusada, Tatsuya Tsukuda, and Yuichi Negishi. Au_{25} Clusters Containing Unoxidized Tellurolates in the Ligand Shell, J. Phys. Chem. Lett. 2014, 5, 2072−2076.
34. Li, Y.; Silverton, L. C.; Haasch, R.; Tong, Y. Y. Alkanetelluroxide-Protected Gold Nanoparticles. Langmuir 2008, 24, 7048−7053.

XYZ-coordinates for the optimized structures
$Au_{20}(S_2C_8H_{18})$, ^1A, PBEPBE/Lanl2dz

79	-0.031018	1.887837	0.302660
79	2.563956	1.325869	1.283045
79	1.478275	-1.233673	2.518460
79	0.290515	-3.102489	0.958066
79	-2.409027	-2.591722	-0.237866
79	-0.944061	-4.889061	-0.745534
79	0.021644	-2.636669	-2.013214
79	-1.371753	-0.212635	-1.454440
79	-3.675828	-0.249926	0.265635
79	-2.359323	1.814819	2.033915
79	0.095188	1.358071	3.088926
79	2.612302	0.772266	4.026101
79	-2.620318	2.301432	-0.886134
79	-4.811400	2.206097	0.794303
79	-0.481014	2.276567	-2.538401
79	1.794799	2.095519	-4.107802
79	2.215860	1.787166	-1.393266
79	1.484179	-0.822054	-0.342194
79	-1.129324	-0.696578	1.505239
79	0.932167	-0.328467	-3.117850
16	4.291089	-1.482084	-0.556845
16	5.173275	0.078831	0.926163
6	4.445949	-3.068755	0.499293
6	3.847508	-4.234338	-0.309421
1	5.515995	-3.213154	0.722796
1	3.876195	-2.893299	1.428451
6	3.821155	-5.541016	0.524766
1	2.810018	-3.983814	-0.609982
1	4.430142	-4.396448	-1.237621
6	3.189925	-6.711009	-0.259501
1	4.851967	-5.807388	0.833721
1	3.242693	-5.364182	1.453688
1	3.179591	-7.636079	0.344938
1	2.144625	-6.476483	-0.538801
1	3.750228	-6.918818	-1.190322
6	6.035871	1.221355	-0.344197
6	6.418457	2.535108	0.358686
1	6.912617	0.676791	-0.732370
1	5.308891	1.402023	-1.155146
6	7.099693	3.519541	-0.626730
1	5.507493	3.008386	0.775519
1	7.103202	2.333633	1.206368
6	7.473893	4.852698	0.055401
1	8.008949	3.048800	-1.051352
1	6.414934	3.715110	-1.476284
1	7.959171	5.542790	-0.658033
1	6.575830	5.357774	0.457150
1	8.173421	4.687585	0.896031

$Au_{20}(S_2C_8H_{18})$, ^3A, PBEPBE/Lanl2dz

79	-0.175188	-1.779025	-0.623009
79	2.491096	-1.243027	-1.442982
79	1.650100	1.562661	-2.098682
79	0.523630	3.198251	-0.291826
79	-2.281683	2.680882	0.651266
79	-0.739024	4.740118	1.637622
79	0.090036	2.221319	2.417833
79	-1.456838	0.055529	1.458527
79	-3.642259	0.553070	-0.341155
79	-2.367741	-1.251467	-2.360266
79	0.172396	-0.772253	-3.225999
79	2.692414	-0.095170	-4.033030
79	-2.839516	-2.224911	0.417994
79	-4.899298	-1.695295	-1.361551
79	-0.733755	-2.636436	2.062705
79	1.505857	-2.892367	3.671753
79	1.998819	-2.044660	1.102927
79	2.123720	0.971177	0.791735
79	-1.007880	1.061904	-1.329583
79	0.831153	-0.271158	3.095878
16	4.639443	0.971267	0.813946
16	5.007706	-1.227202	-1.229916
6	5.039324	2.355612	-0.441981
6	4.615619	3.740872	0.074763
1	6.129521	2.293956	-0.608675
1	4.512035	2.100126	-1.378152
6	4.892399	4.854048	-0.965669
1	3.531690	3.723700	0.309408
1	5.147023	3.970651	1.019474
6	4.445628	6.243007	-0.460432
1	5.973711	4.875285	-1.210423
1	4.357351	4.612466	-1.906609
1	4.643568	7.027167	-1.213666
1	3.361213	6.250308	-0.239902
1	4.980309	6.520216	0.467352
6	5.402102	-2.570997	0.065888
6	4.958370	-3.974018	-0.378371
1	6.497015	-2.511019	0.207343
1	4.905267	-2.263630	1.004202
6	5.239576	-5.040005	0.709505
1	3.872000	-3.956090	-0.600764
1	5.475289	-4.252945	-1.317765
6	4.785952	-6.449382	0.271431
1	6.322643	-5.053587	0.947028
1	4.712780	-4.754234	1.643038
1	4.989102	-7.198965	1.057660
1	3.700514	-6.466081	0.059300
1	5.313555	-6.769360	-0.646496

Au$_{20}$(Se$_2$C$_8$H$_{18}$), ^{1}A, PBEPBE/Lanl2dz

79	-0.129096	-1.862781	-0.371143
79	2.487564	-1.328364	-1.289056
79	1.413982	1.285702	-2.446075
79	0.232110	3.112870	-0.834415
79	-2.488427	2.606879	0.307272
79	-0.998377	4.866210	0.907839
79	-0.074156	2.560982	2.110848
79	-1.462288	0.174865	1.427171
79	-3.775773	0.298263	-0.283197
79	-2.461904	-1.719136	-2.113218
79	0.005691	-1.256403	-3.131996
79	2.531840	-0.667524	-4.028959
79	-2.745965	-2.300894	0.774183
79	-4.932073	-2.124093	-0.908436
79	-0.635865	-2.359925	2.457920
79	1.620629	-2.255381	4.068493
79	2.071443	-1.873416	1.368998
79	1.465539	0.819102	0.415512
79	-1.216403	0.760194	-1.496575
79	0.795261	0.207324	3.148531
34	4.239138	1.407459	0.697106
34	5.138496	-0.308588	-0.957209
6	4.408946	3.051721	-0.488744
6	3.811626	4.259034	0.255995
1	5.481433	3.177460	-0.713807
1	3.841497	2.828374	-1.409400
6	3.823838	5.528499	-0.635317
1	2.763851	4.037501	0.543226
1	4.375995	4.457577	1.188870
6	3.192253	6.740707	0.081874
1	4.865601	5.766485	-0.930292
1	3.265045	5.318441	-1.569094
1	3.203842	7.636091	-0.565590
1	2.139597	6.531312	0.352326
1	3.738674	6.986190	1.011767
6	5.877529	-1.616251	0.418652
6	6.111483	-2.979192	-0.255240
1	6.805091	-1.172306	0.817493
1	5.114535	-1.690991	1.213628
6	6.655250	-4.018657	0.759413
1	5.157465	-3.349554	-0.679436
1	6.829691	-2.879367	-1.093764
6	6.886941	-5.398154	0.106341
1	7.603845	-3.648120	1.197100
1	5.934883	-4.119489	1.595892
1	7.261357	-6.129495	0.845025
1	5.947528	-5.797702	-0.318829
1	7.628437	-5.333638	-0.711646

$Au_{20}(Se_2C_8H_{18})$, ^3A, PBEPBE/Lanl2dz

79	0.853958	-0.850317	0.895025
79	2.046030	-1.874307	-1.508180
79	-0.606429	-1.811473	-2.765214
79	-2.220870	0.376160	-2.799185
79	-3.548693	1.307340	-0.258936
79	-3.847593	2.616590	-2.666063
79	-1.339007	2.902956	-1.539407
79	-0.996469	1.599765	0.986196
79	-3.078863	-0.005756	2.078164
79	-1.278658	-2.417529	2.094513
79	-0.213804	-3.312863	-0.214735
79	0.741858	-4.176671	-2.636770
79	-0.337224	0.087117	3.334908
79	-2.439756	-1.384458	4.379273
79	1.628696	1.556177	2.173450
79	3.425839	3.279819	1.064740
79	2.975969	0.811030	-0.355459
79	0.391797	0.575784	-1.612473
79	-1.952152	-1.048974	-0.335613
79	1.070594	3.071337	-0.327623
34	5.114209	0.396256	-1.760312
34	4.493967	-2.669459	-1.136735
6	6.608189	0.730060	-0.419915
6	6.767964	2.217393	-0.064188
1	6.384615	0.122332	0.473757
1	7.516404	0.329322	-0.905924
6	7.891562	2.455954	0.974477
1	6.972626	2.807429	-0.979047
1	5.806204	2.598935	0.349069
6	8.000960	3.941576	1.380185
1	7.695808	1.838954	1.874751
1	8.858659	2.109858	0.557006
1	8.809636	4.099073	2.116523
1	8.212174	4.580249	0.502137
1	7.055946	4.296343	1.835252
6	4.588797	-2.382477	0.872545
6	3.654784	-3.313325	1.663644
1	5.650284	-2.543135	1.137381
1	4.327297	-1.320223	1.038852
6	3.688296	-3.011408	3.183445
1	2.616632	-3.184205	1.295998
1	3.927993	-4.372136	1.484072
6	2.684618	-3.883772	3.968676
1	4.713689	-3.172273	3.574195
1	3.448167	-1.940836	3.345147
1	2.717541	-3.657220	5.050111
1	1.651187	-3.703382	3.615499
1	2.903461	-4.960496	3.839511

Au$_{20}$(Se$_2$C$_8$H$_{18}$)$_2$, ^{1}A, PBEPBE/Lanl2dz

79	-0.775869	0.572568	-1.827204
79	0.491300	2.897125	-0.823678
79	-1.265894	2.665305	1.662294
79	-1.160727	0.431118	3.193325
79	-2.349652	-2.137610	2.191604
79	-1.021139	-1.930167	4.616300
79	0.644038	-1.856677	2.413986
79	-0.657648	-1.952695	-0.125362
79	-3.515761	-2.214123	-0.253939
79	-3.659763	0.274248	-1.919910
79	-2.598539	2.598210	-1.015954
79	-1.365906	4.843853	-0.017166
79	-1.812708	-1.942480	-2.728846
79	-4.592060	-2.180024	-2.783780
79	0.899264	-1.718204	-2.525715
79	3.605979	-1.461792	-2.211507
79	2.032369	0.763445	-1.571690
79	0.698315	0.654912	1.008131
79	-2.485685	0.293033	0.674195
79	2.160200	-1.679719	0.165832
34	2.981354	2.103044	2.050283
34	2.265689	4.350949	1.155634
6	2.447563	2.317921	4.002738
6	2.667644	0.971722	4.715525
1	3.062254	3.130766	4.424175
1	1.382121	2.604101	3.997270
6	2.151805	1.008828	6.177741
1	2.125965	0.170577	4.172813
1	3.743400	0.703987	4.710552
6	2.351998	-0.346074	6.889678
1	2.672576	1.811144	6.738453
1	1.074042	1.267256	6.172139
1	1.966062	-0.314478	7.924863
1	1.816586	-1.151750	6.351398
1	3.422638	-0.621669	6.933379
6	4.065742	5.041718	0.481377
6	3.817210	6.108024	-0.600561
1	4.607952	5.445494	1.352974
1	4.607996	4.170620	0.076856
6	5.148236	6.671867	-1.162327
1	3.225590	5.667353	-1.427000
1	3.219386	6.944355	-0.185116
6	4.913377	7.733365	-2.258655
1	5.740730	7.112572	-0.335570
1	5.751258	5.837250	-1.574726
1	5.870574	8.121560	-2.651555
1	4.345514	7.308258	-3.107084
1	4.337507	8.590903	-1.864205
34	5.766779	-2.375867	0.520616
34	6.306819	-1.612760	-1.809252
6	7.622498	-2.997194	1.087628
1	7.503070	-3.139689	2.179773
6	8.092238	-4.282932	0.392882
1	8.313825	-2.152672	0.923159

6	9.476990	-4.746057	0.912214
1	7.347313	-5.086180	0.553744
1	8.149937	-4.115609	-0.701322
6	9.959371	-6.036110	0.213541
1	10.220049	-3.937972	0.755773
1	9.421195	-4.915718	2.006490
1	10.945433	-6.355080	0.597044
1	9.247529	-6.866133	0.378587
1	10.053116	-5.885876	-0.878169
6	6.501125	0.393261	-1.469328
6	6.371944	1.165728	-2.791936
1	5.697511	0.650692	-0.759622
1	7.486607	0.531491	-0.992690
6	6.490773	2.694979	-2.564672
1	7.153719	0.844094	-3.508910
1	5.389406	0.945403	-3.254952
6	6.369862	3.489908	-3.882555
1	5.693919	3.015755	-1.863324
1	7.459701	2.923522	-2.076564
1	6.449669	4.577817	-3.702588
1	7.167874	3.206790	-4.593712
1	5.396939	3.299291	-4.372173

$Au_{20}(Te_2C_8H_{18})_2$, ^1A, PBEPBE/Lanl2dz

79	0.956534	-1.594500	1.058235
79	-0.808208	-0.138019	2.827364
79	0.932572	2.338543	2.296208
79	1.112378	3.319141	-0.223857
79	2.692513	1.845204	-2.311570
79	1.273021	4.171543	-2.841762
79	-0.272910	1.914648	-2.479787
79	1.069867	-0.538568	-1.940110
79	3.946416	-0.459143	-1.671335
79	3.854131	-1.460905	1.174740
79	2.360397	-0.153102	3.024238
79	0.730308	1.186139	4.784262
79	2.417767	-2.899790	-1.032784
79	5.208228	-2.756319	-0.852150
79	-0.300734	-2.933611	-1.230146
79	-3.013813	-2.828140	-1.538661
79	-1.857527	-1.573314	0.716951
79	-0.431114	0.940987	0.218851
79	2.643540	0.986288	0.413683
79	-1.792822	-0.287055	-1.957864
52	-3.279438	2.344797	1.199771
52	-3.329375	1.052470	3.772961
6	-5.003213	3.716329	1.513602
6	-5.016654	4.809044	0.426323
1	-5.922031	3.103174	1.501633
1	-4.870715	4.145362	2.522336
6	-6.211803	5.781405	0.606608
1	-4.071569	5.386581	0.458909
1	-5.075728	4.354344	-0.583840
6	-6.243968	6.874831	-0.483122
1	-7.159393	5.205875	0.587649

1	-6.148423	6.252385	1.607696
1	-7.099723	7.558528	-0.337993
1	-5.319572	7.481538	-0.464993
1	-6.334209	6.430173	-1.492664
6	-4.621060	-0.684948	3.341523
6	-4.059348	-1.974124	3.968488
1	-5.623483	-0.429918	3.728976
1	-4.659022	-0.759107	2.238935
6	-4.944402	-3.199632	3.623991
1	-3.032938	-2.148500	3.587653
1	-3.986736	-1.877910	5.071099
6	-4.377102	-4.510253	4.211723
1	-5.973889	-3.033211	4.002168
1	-5.016693	-3.289740	2.520961
1	-5.016272	-5.374144	3.952990
1	-3.362093	-4.709166	3.820990
1	-4.310039	-4.457330	5.314476
52	-4.695790	0.017419	-0.880134
52	-5.611020	-2.324897	-2.506914
6	-6.171611	1.498232	-1.659171
1	-6.358700	2.178910	-0.807038
6	-5.673785	2.251991	-2.903762
1	-7.094493	0.923892	-1.855579
6	-6.692150	3.316214	-3.385616
1	-4.703455	2.742312	-2.686359
1	-5.478726	1.532964	-3.724078
6	-6.191693	4.083115	-4.629372
1	-7.659094	2.824459	-3.613931
1	-6.892215	4.033000	-2.561751
1	-6.924547	4.843722	-4.954320
1	-5.236100	4.599651	-4.419823
1	-6.020059	3.394311	-5.477219
6	-6.763450	-3.312498	-0.898100
6	-6.145711	-4.649872	-0.453276
1	-6.801517	-2.581354	-0.070981
1	-7.782554	-3.444400	-1.305286
6	-6.973297	-5.326349	0.668779
1	-6.070503	-5.341664	-1.316091
1	-5.109081	-4.478263	-0.097307
6	-6.343663	-6.658690	1.131913
1	-7.055340	-4.634125	1.531295
1	-8.006280	-5.507264	0.308668
1	-6.941798	-7.128175	1.933954
1	-6.276325	-7.378335	0.294844
1	-5.319957	-6.498589	1.519281

$Au_{20}(Te_2C_8H_{18})$, ^1A, PBEPBE/Lanl2dz

79	1.085023	1.553430	0.996370
79	-1.658384	2.231925	1.138465
79	-2.149658	1.176466	-1.636725
79	-1.470497	-1.373820	-2.242730
79	1.395634	-2.217315	-2.372169
79	-0.704968	-3.981464	-2.744390
79	-0.359515	-3.160368	-0.128057
79	1.715865	-1.287259	0.318767

79	3.341668	-0.407485	-1.874865
79	2.671059	2.406300	-1.230502
79	0.081298	3.198838	-1.123020
79	-2.588545	3.827887	-0.923496
79	3.800089	0.613841	0.892708
79	5.279281	1.469214	-1.281273
79	2.238501	-0.243552	2.929494
79	0.485765	-1.138999	4.872900
79	-0.624883	0.609963	3.041462
79	-1.586411	-0.635620	0.593167
79	0.598702	0.493659	-1.814542
79	0.032109	-2.234210	2.383585
52	-4.291735	-1.223968	1.223597
52	-5.010849	1.261210	0.019553
6	-4.690298	-2.515801	-0.521353
6	-4.224145	-3.958669	-0.258333
1	-5.777012	-2.454370	-0.710755
1	-4.138452	-2.051041	-1.359249
6	-4.487128	-4.871054	-1.484723
1	-3.135940	-3.964274	-0.040718
1	-4.737134	-4.383268	0.628786
6	-3.964566	-6.306723	-1.261563
1	-5.573938	-4.894588	-1.703947
1	-3.990908	-4.429614	-2.373175
1	-4.156680	-6.942600	-2.143866
1	-2.873588	-6.302700	-1.075457
1	-4.454424	-6.780213	-0.390233
6	-7.226371	1.033871	0.080008
6	-7.881486	2.283808	-0.541080
1	-7.478126	0.118466	-0.483175
1	-7.504769	0.906739	1.140858
6	-9.430078	2.190367	-0.525865
1	-7.571439	3.192176	0.014403
1	-7.540501	2.413082	-1.588618
6	-10.095720	3.441866	-1.139597
1	-9.745613	1.286451	-1.084905
1	-9.777630	2.055486	0.518229
1	-11.197252	3.354886	-1.126175
1	-9.821948	4.354493	-0.577466
1	-9.779978	3.585225	-2.190091

$Au_{20}(Te_2C_8H_{18})$, ^3A, PBEPBE/Lanl2dz

79	1.383576	-0.283878	0.907955
79	2.419583	2.285120	0.314135
79	1.276929	1.244111	-2.323011
79	-1.386341	0.747955	-2.599112
79	-2.392508	-2.030075	-1.861763
79	-3.940256	-0.159246	-3.188558
79	-3.409501	0.396897	-0.538362
79	-1.648995	-1.277602	0.815048
79	-0.743364	-3.696320	-0.489240
79	2.133978	-2.926213	-0.087567
79	3.117305	-0.767714	-1.401296
79	4.039180	1.623001	-2.369114
79	0.321369	-2.773028	2.169951

79	1.014127	-5.198023	1.033966
79	-0.583932	-0.488463	3.277385
79	-1.312758	1.947697	4.311856
79	0.550560	1.969420	2.269472
79	-0.463721	2.366457	-0.455597
79	0.319875	-1.321863	-1.609215
79	-2.367032	1.337096	1.780859
52	-2.369470	3.927784	0.723845
52	4.455472	3.793645	-0.762480
6	-3.872509	3.902362	-0.900118
6	-5.283726	3.521262	-0.424894
1	-3.837284	4.928455	-1.314379
1	-3.488410	3.199185	-1.659594
6	-6.302086	3.518319	-1.593742
1	-5.255817	2.509591	0.032550
1	-5.629879	4.217277	0.366126
6	-7.714600	3.094186	-1.137620
1	-6.342617	4.527254	-2.052268
1	-5.942267	2.825439	-2.381919
1	-8.426959	3.098392	-1.982618
1	-7.700677	2.073491	-0.711253
1	-8.105316	3.778595	-0.361657
6	6.064428	2.958637	0.503042
6	5.985022	1.434452	0.706725
1	7.008351	3.258626	0.012082
1	5.973111	3.500306	1.462497
6	7.102849	0.911672	1.642324
1	4.995604	1.163285	1.128008
1	6.054012	0.923282	-0.275284
6	7.042498	-0.623475	1.812316
1	8.093586	1.203657	1.237444
1	7.008275	1.399652	2.633487
1	7.835740	-0.981933	2.493368
1	6.066823	-0.939057	2.228765
1	7.172509	-1.135401	0.840032

$Au_{20}(S_2O_2C_8H_{18})_2$, ^3A, PBEPBE/Lanl2dz

6	1.697941	5.571360	-1.268003
6	2.955159	5.571834	-0.372051
6	3.558400	4.153743	-0.214721
6	4.817872	4.159971	0.665199
16	5.533036	2.414477	0.959032
16	5.595825	-1.232484	-1.018963
6	5.584658	-2.806835	0.080750
6	4.333225	-3.673044	-0.098204
6	4.385873	-4.945675	0.782288
6	3.111543	-5.804109	0.623990
79	0.750075	1.417923	-0.424345
79	0.162303	2.448776	2.183450
79	2.564334	0.679311	1.731907
79	1.809946	1.659560	4.245280
79	0.191396	-0.334680	3.300867
79	-1.469635	-2.208200	2.266566
79	-3.176368	-4.027718	1.084342
79	-0.993069	-3.264836	-0.448458

79	-1.210757	-0.647589	-1.593687
79	1.061464	-2.353390	-1.958937
79	3.238497	-1.321037	-3.307471
79	1.084552	0.344919	-3.043553
79	-1.022544	2.021036	-2.575500
79	-1.510902	3.102000	0.158581
79	-1.679093	0.464742	1.267564
79	-3.384721	-1.474838	0.067808
79	-3.400166	1.054016	-0.933655
79	-3.227078	3.610293	-1.931270
79	3.029301	-0.292214	-0.763551
79	0.794002	-1.333476	0.711327
1	4.621362	4.566903	1.672807
1	5.647118	4.723564	0.197217
1	3.798185	3.739613	-1.214104
1	2.794737	3.486900	0.233744
1	2.696140	5.971415	0.629478
1	3.717818	6.254925	-0.799058
1	1.281751	6.590122	-1.372605
1	1.929794	5.191564	-2.280935
1	0.908820	4.922792	-0.841695
1	6.507337	-3.349417	-0.192967
1	5.680154	-2.400468	1.104732
1	3.431749	-3.083229	0.163144
1	4.234566	-3.962338	-1.162081
1	5.276918	-5.547236	0.510820
1	4.507533	-4.656893	1.845809
1	3.163038	-6.713726	1.249364
1	2.211068	-5.235387	0.922569
1	2.974556	-6.121253	-0.426970
8	4.464946	1.806664	2.187726
8	5.357063	-1.891217	-2.615202

$Au_{20}(Se_2O_2C_8H_{18})$, ^3A, PBEPBE/Lanl2dz

6	1.853778	5.392562	-1.634728
6	3.053506	5.396312	-0.663464
6	3.646906	3.979898	-0.456588
6	4.853136	4.001111	0.496613
34	5.620430	2.165375	0.864149
34	5.380136	-1.683483	-0.836389
6	5.120082	-3.314999	0.351667
6	3.810446	-4.060017	0.071878
6	3.664049	-5.333691	0.941702
6	2.323949	-6.053521	0.674875
79	0.747255	1.320912	-0.495534
79	0.220426	2.549693	2.040795
79	2.486305	0.582702	1.716333
79	1.788717	1.759701	4.163167
79	0.031595	-0.158760	3.318300
79	-1.758607	-1.961030	2.379875
79	-3.588289	-3.716325	1.286462
79	-1.343377	-3.209531	-0.263163
79	-1.357656	-0.654864	-1.559114
79	0.781830	-2.542479	-1.807674
79	3.041838	-1.760634	-3.192887

79	1.021635	0.068074	-3.044409
79	-0.959532	1.925217	-2.693160
79	-1.385625	3.203388	-0.034639
79	-1.763070	0.658706	1.226843
79	-3.598426	-1.218323	0.122806
79	-3.417100	1.241599	-1.025779
79	-3.042292	3.713888	-2.171357
79	2.899484	-0.567669	-0.713513
79	0.575134	-1.350811	0.798147
1	4.590987	4.400201	1.492883
1	5.696277	4.585538	0.081110
1	3.944142	3.558176	-1.437324
1	2.858538	3.315774	-0.048399
1	2.735115	5.804138	0.317166
1	3.841945	6.074884	-1.048533
1	1.447179	6.412368	-1.768888
1	2.147050	5.008187	-2.630590
1	1.037225	4.747792	-1.254965
1	6.005850	-3.947040	0.156544
1	5.172342	-2.908439	1.378887
1	2.951502	-3.386409	0.266512
1	3.766990	-4.337396	-0.999387
1	4.506860	-6.023649	0.734186
1	3.730843	-5.060699	2.014273
1	2.226419	-6.960486	1.298877
1	1.467856	-5.389870	0.899482
1	2.241945	-6.358677	-0.385300
8	4.449276	1.584922	2.149007
8	5.127885	-2.429625	-2.495341

$Au_{20}(Te_2O_2C_8H_{18})$, ^3A, PBEPBE/Lanl2dz

6	1.641435	5.508171	-1.734513
6	2.835507	5.533518	-0.756275
6	3.492106	4.138655	-0.591283
6	4.697633	4.189911	0.366648
52	5.639999	2.245531	0.714494
52	5.275045	-1.962340	-0.713884
6	4.831604	-3.650932	0.620363
6	3.501933	-4.349072	0.300472
6	3.229370	-5.547901	1.242271
6	1.868311	-6.210596	0.939804
79	0.681581	1.233368	-0.615258
79	0.194468	2.707286	1.801894
79	2.401738	0.637288	1.649418
79	1.742423	2.062579	3.982448
79	-0.082695	0.140249	3.317821
79	-1.932897	-1.679324	2.549842
79	-3.820067	-3.467434	1.616943
79	-1.556567	-3.178205	0.036900
79	-1.488440	-0.757209	-1.490044
79	0.584241	-2.726100	-1.569521
79	2.860661	-2.167926	-3.038439
79	0.910541	-0.264145	-3.042894
79	-1.004374	1.694390	-2.853783
79	-1.391671	3.223722	-0.324326

79	-1.852081	0.819695	1.162482
79	-3.748187	-1.091151	0.225973
79	-3.481666	1.244730	-1.143977
79	-3.028563	3.590247	-2.506507
79	2.777887	-0.739016	-0.672872
79	0.422640	-1.294278	0.916156
1	4.409934	4.546484	1.373020
1	5.502288	4.842277	-0.024295
1	3.808715	3.763923	-1.585324
1	2.732672	3.426566	-0.209115
1	2.494397	5.894958	0.235319
1	3.596469	6.258132	-1.113687
1	1.199733	6.515217	-1.849925
1	1.951214	5.153744	-2.736106
1	0.846213	4.828355	-1.370743
1	5.692037	-4.337137	0.506263
1	4.846863	-3.216378	1.637754
1	2.667938	-3.624811	0.391580
1	3.507154	-4.700225	-0.750054
1	4.041638	-6.294887	1.138398
1	3.246621	-5.200604	2.294781
1	1.675800	-7.057381	1.622817
1	1.041334	-5.483688	1.054062
1	1.835919	-6.594874	-0.096903
8	4.392920	1.556295	2.067413
8	4.957647	-2.834880	-2.449000
